# Health Monitoring via Heart, Breath, and Korotkoff Sounds by Wearable Piezoelectret Patches

**DOI:** 10.1002/advs.202301180

**Published:** 2023-08-21

**Authors:** Liuyang Han, Weijin Liang, Qisen Xie, JingJing Zhao, Ying Dong, Xiaohao Wang, Liwei Lin

**Affiliations:** ^1^ Tsinghua Shenzhen International Graduate School Tsinghua University 518055 Shenzhen China; ^2^ Department of mechanical engineering University of California Berkeley Berkeley USA

**Keywords:** audio physiological signals, blood pressure measurement, breath sounds classification, component separation, piezoelectret sensor, wearable systems

## Abstract

Real‐time monitoring of vital sounds from cardiovascular and respiratory systems via wearable devices together with modern data analysis schemes have the potential to reveal a variety of health conditions. Here, a flexible piezoelectret sensing system is developed to examine audio physiological signals in an unobtrusive manner, including heart, Korotkoff, and breath sounds. A customized electromagnetic shielding structure is designed for precision and high‐fidelity measurements and several unique physiological sound patterns related to clinical applications are collected and analyzed. At the left chest location for the heart sounds, the S1 and S2 segments related to cardiac systole and diastole conditions, respectively, are successfully extracted and analyzed with good consistency from those of a commercial medical device. At the upper arm location, recorded Korotkoff sounds are used to characterize the systolic and diastolic blood pressure without a doctor or prior calibration. An Omron blood pressure monitor is used to validate these results. The breath sound detections from the lung/ trachea region are achieved a signal‐to‐noise ration comparable to those of a medical recorder, BIOPAC, with pattern classification capabilities for the diagnosis of viable respiratory diseases. Finally, a 6×6 sensor array is used to record heart sounds at different locations of the chest area simultaneously, including the Aortic, Pulmonic, Erb's point, Tricuspid, and Mitral regions in the form of mixed data resulting from the physiological activities of four heart valves. These signals are then separated by the independent component analysis algorithm and individual heart sound components from specific heart valves can reveal their instantaneous behaviors for the accurate diagnosis of heart diseases. The combination of these demonstrations illustrate a new class of wearable healthcare detection system for potentially advanced diagnostic schemes.

## Graphical abstract

Flexible piezoelectret patches are developed to examine audio physiological signals from cardiovascular and respiratory systems, including heart, Korotkoff, and breath sounds. A customized electromagnetic shielding structure is designed for precision and high‐fidelity measurements and several unique physiological sound patterns have been collected and analyzed. This work illustrates a new class of wearable healthcare detection system for potentially advanced diagnostic schemes.

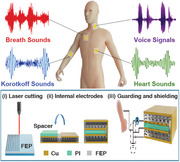

## 1 Introduction

A wearable mobile platform to continuously monitor a variety of body vital signs is attractive for next‐generation diagnostic and therapeutic applications. For example, recent advances in skin electronics have shown various cutting‐edge detection schemes to gather results in the forms of sound, temperature, pressure, impedance, and chemistry. ^[^ ^1^, ^2^, ^3^, ^4^ ^]^ Specifically, acoustic signals are generated during different physiological activities, including breathing, swallowing, heart beating, and talking ^[^ ^5^, ^6^, ^7^ ^]^ and these signals contain important pathological data from the cardiovascular and respiratory systems. Previously, heart sounds or phonocardiography (PCG) have been used for the diagnosis of congestive heart failure and myocardial ischemia by analyzing the systolic and diastolic interval or amplitude.^[^ ^8^, ^9^, ^10^ ^]^ The detection of pulse waves at different body locations has been used to estimate the artery stiffness and blood pressure.^[^ ^11^, ^12^, ^13^ ^]^ Abnormal breath sounds, such as wheezes, rhonchi, and crackles, usually indicate pulmonary disorder^[^ ^14^ ^]^ while regional varieties in the breath sound intensity are related to patients with chronic obstructive pulmonary diseases.^[^ ^15^ ^]^ Some researchers have also demonstrated fascinating applications by detecting heart and breath sounds in the recognition of emotion, identity and physiological conditions.^[^ ^16^, ^17^, ^18^ ^]^

In general, it is very challenging to accurately record acoustic signals from human body due to complex frequency bands, rhythms, and subtle intensities. Sensors for pulse detections have been widely investigated by means of piezoresistive, capacitive, piezoelectric, and triboelectric mechanisms.^[^ ^19^, ^20^, ^21^, ^22^, ^23^ ^]^ For audio physiological signals, Gupta et al. have utilized a high‐precision fabrication technique for capacitive sensors with a linear response range of from 0 to 12 kHz.^[^ ^24^ ^]^ Rogers et al. have leveraged the commercial MEMS accelerometer with stretchable circuits to monitor PCG, seismocardiography (SCG), and pulses.^[^ ^5^, ^25^ ^]^ Cotur et al. have demonstrated vital sign measurements using a commercial microphone with flexible packages.^[^ ^26^ ^]^ These MEMS‐based systems require rather complex fabrication processes to construct sensors of limited conformability or flexibility. On the other hand, Nayeem et al. and Yan et al. have utilized fiber‐based flexible structures to achieve a wide working frequency range (hundreds of Hertz) for epidermal sensors.^[^ ^6^, ^27^ ^]^ Ha et al. have employed a 28 µm thick piezoelectric polymer system to detect SCG signals.^[^ ^17^ ^]^ These flexible sensors have excellent conformability but limited sensitivity and often focus on a single vital sign instead of a combination of physiological signals from both the cardiovascular and respiratory system.

This paper presents a flexible sensing system to comprehensively detect both cardiovascular and respiratory signals based on a folded double‐layer piezoelectret sensor to record a wide range of vital signs, including pulses, heart sounds, Korotkoff sounds, breath sounds, and human voices. Piezoelectric and piezoelectret sensors take the advantages of simple construction and high sensitivity and have been extensively studied.^[^ ^28^, ^29^ ^]^ The FEP‐Air‐FEP sandwich structure with a symmetrical design exhibits an excellent dynamic sensitivity of 591 pC kPa^−1^ in the range of 0–8 kPa; a minimum pressure resolution of less than 5 Pa; a wide frequency bandwidth of 0–600 Hz with a frequency resolution <0.1 Hz; and a long stable operation period of more than 1.1 million cycles. A protection layer is utilized for the excellent electromagnetism shielding by suppressing key 50 Hz electromagnetic power noises in the human body. Several experimental demos have been conducted. First, heart sounds are measured and used to analyze the S2 physiological split phenomenon during the inspiration process. Instantaneous cardiac activities are found to be consistent with those from ECG references and medical physiological records. Second, quantified Korotkoff sounds are recorded at the upper arm area and analyzed to demonstrate the noninvasive blood pressure assessment. Third, breath sounds are detected and examined at the lung/trachea region for the recognition of both breath pattern and human identity. Forth, a large‐area, 6×6 sensing system is utilized at the chest region to record heart sounds from the operation of four heart valves and the ICA (independent component analysis) algorithm is developed to extract data related to individual heart valves for advanced diagnosis of heart diseases.

## 2 Results and Discussion

### 2.1 Performance Characterizations and Physiological Signal Measurements

**Figure 1:**
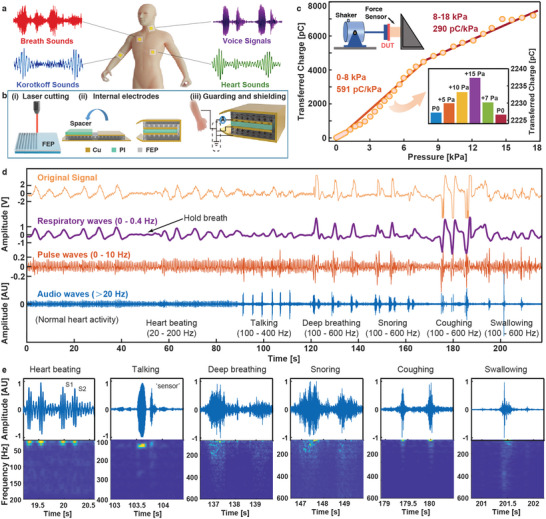
Performance characterizations and physiological signal measurements of a prototype piezoelectret sensor. a) Illustration of the piezoelectret sensor patches at different body locations. b) The schematic diagram of a prototype sensor, showing key aspects such as i) laser processing for FEP grooves; ii) internal electrodes for electromechanical conversion; iii) external electrode for guarding and shielding structure. c) Result of transferred charges versus applied pressure showing the dynamic sensitivity variations between the applied pressure of 0–18 kPa at a constant frequency of 220 Hz. Inset (top): experimental setup for characterizing the dynamic sensitivity of the piezoelectret sensor. Inset (bottom): measured transferred charges as the applied pressure increases from the initial state (P0) to extra + 5 Pa, + 10 Pa, and + 15 Pa, and decreases from extra + 15 Pa, + 7 Pa to the initial state (P0), where P0 is the initial reference pressure chosen arbitrarily in the low linearity region. d) Recorded data from the neck position of a volunteer including: (top) the original data; (bottom) the respiratory wave (0–0.4 Hz), the pulse wave (0–10 Hz), and the audio wave (> 20 Hz) by processing the original data with filters of various frequency ranges. The volunteer is asked to perform activities sequentially to record the normal heart beating, talking, deep breathing, snoring, coughing, and swallowing. e) Enlarged time series (top) and STFT (short‐time Fourier transform) spectrograms (bottom) corresponding to different physiological activities.

**Figure** **1**a illustrates the piezoelectret sensor patches placed at different body locations for breath, Korotkoff, voice, and heart sounds and the typical recording patterns. The fabrication process is schematically depicted in Figure S1, Supporting Information. Specifically, parallel grooves on the surfaces of two FEP films are constructed by a laser cutting process in Figure 1b‐i. A hot‐pressing process is followed to bond the two FEP films together with the two groove surfaces facing each other perpendicularly to form the crisscross air cavity (Figure S2, Supporting Information). A copper tape is then attached as the external electrode on one side and a Corona charging process with a high‐electric field is used to generate electric dipoles. Subsequently, two separated copper tapes (internal electrodes) are attached on the other side. The FEP strip is folded with a polyimide tape as the spacer layer to insulate the two internal electrodes as shown in Figure 1b‐ii. The process is completed as shown in Figure 1b‐iii, in which the external copper tape is used as the ground electrode to wrap around the entire sensor as well as the electrical shielding structure to ensure the accurate detection of physiological signals (Figures S3 and S4, Supporting Information).

Electric dipoles formed during the Corona charging process ensure the piezoelectric‐like behavior of the sensor. The FEP cavity captures the positive and negative ions after the air in the closed crisscross cavity is ionized by the high electric field, which further induced free charges on the internal electrodes. The amount of these free charges fluctuates periodically in response to the applied pressure, resulting in alternating current outputs in the external circuit (Figure S5, Supporting Information). The piezoelectret sensor can achieve a high sensitivity because of the large porosity of the FEP electret with crisscross cavities.^[^ ^29^ ^]^ A pressure test is designed (Figure S6, Supporting Information) for the sensitivity characterization, in which the modal shaker and force sensor are used to apply controlled pressure on the device under test (DUT) and the outputs are amplified by a current preamplifier (Stanford SR570). The sensor exhibits a dynamic sensitivity with two distinct linear regions, which is consistent with previous works based on piezoelectric materials.^[^ ^30^, ^31^ ^]^ The dynamic sensitivity is measured under external pressure inputs with a constant frequency of 220 Hz. Results show an linear relationship of 591 pC kPa^−1^ in the low‐pressure region (<8 kPa), and 290 pC kPa^−1^ in the high‐pressure region of >8 kPa (Figure 1c; Figure S7, Supporting Information). Specifically, under a high pressure, the upper FEP diaphragm experiences a large deformation to physically touch the lower FEP film and reduce the possible further deformation resulting in the slightly lower sensitivity as described in our previous work.^[^ ^29^ ^]^ By comparison, the prototype sensor in this paper expands the linear operation range to 8 kPa due to the thicker folded double‐layer structure. The corresponding transferred charges under different applied pressures are found in Figure S8 (Supporting Information). Furthermore, the prototype sensor exhibits a sensitive resolution for the dynamic pressure changes of about 5 Pa as shown in the inset of Figure 1c. In this test, an arbitrary pressure P0 is applied as the reference and the influence of the relative pressure change (∆*P*) on the output charge (∆*Q*) is evaluated. Experimental results show that slight fluctuations in the applied pressure can cause significant variations on the transferred charges with good repeatability and linearity (Figure S9, Supporting Information). In addition, a portable customized amplification and filter circuit is used to characterize the dynamic sensitivity of the sensor (Figure S10, Supporting Information). This circuit setup contains a 50 Hz notch filter and a 2000 Hz low‐pass filter with an amplitude gain of about 6 V nC^−1^ (Figure S11, Supporting Information) to replace the expensive and bulky current preamplifier SR570 in a wearable manner for the following human experiments. Two linear regions have been obtained under applied pressures with the dynamic sensitivity of 3.33 V kPa^−1^ for pressure <8 kPa and 0.24 V kPa^−1^ for pressure >8 kPa (Figure S12, Supporting Information).

Frequency resolution is also an important parameter. Mixed signals with a frequency difference of 0.1 Hz have been used to drive the modal shaker and signals with a beat frequency period of 10 s can be observed obviously from the sensor's transferred charges (Figure S13, Supporting Information). Fast Fourier transform (FFT) results of the transferred charges show two peaks at 220 Hz and 220.1 Hz, which are consistent with driving signals. Furthermore, the amplitude‐frequency response from a prototype sensor (size: 2 × 2 cm^2^) shows a first‐order resonant frequency of about 740 Hz and a wide working range of 600 Hz (Note S1, Supporting Information) to cover key physiological signals such as voice, heart sound, breath sound, etc.^[^ ^27^, ^32^ ^]^ In this case, three different sensors with the same structure are involved in the characterization of amplitude‐frequency response: FEP (piezoelectret) sensor with Corona charging, FEP sensor without Corona charging, and PET (nonpiezoelectret) sensor without Corona charging, to exclude the influence of electromagnetic interference, triboelectric effect and circuit noise, etc. For the mechanical stability, a repeatability test under a same applied pressure at 220 Hz is conducted for 1.1 million cycles over 5000 s and results show stable transferred charges within ± 2.5% of the mean value during the test (Figure S19, Supporting Information). In this work, the external copper electrode protects the internal FEP film from damages for long‐term stable operations and it also acts as an electrical shielding layer ^[^ ^33^, ^34^ ^]^ to reduce power frequency noises (50 Hz and harmonic frequencies). In actual human experiments, the external shielding layer, the human skin, and the ground electrode of the measuring instrument (or customized circuit) are connected together, forming a guard ring to protect the internal electrodes from the electromagnetic noises, especially the power frequency noises. A comparative experiment has been conducted to show that the device with the external shielding electrode and electrical ground can record distinct pulse waveforms with the lowest noise level (Figure S20, Supporting Information) while other setups fail to obtain clean pulse waveforms.

The good flexibility of the piezoelectret sensor ensures the high‐fidelity acquisition of physiological signals at different curved skin positions (Figure S21, Supporting Information). In the first experiment at the neck position, several physiological signals have been recorded during the nearly four‐minute test. The volunteer is asked to conduct a series of physiological activities, including normal heart beating, talking, deep breathing, snoring, coughing and swallowing. Measured signals are processed by filters of different frequency ranges as shown in Figure 1d. The “original signal” shows the nonfiltered data; the “respiratory waves” is the baseline after applying a low‐pass filter of 0–0.4 Hz; the “pulse waves” is the low‐frequency result after applying a 0–10 Hz low‐pass filter and the deduction of the baseline; the “audio waves” is the high‐frequency result after applying bandpass filters for audio frequency signals, such as: a 20–200 Hz bandpass filter from 0 to 89 s for normal heart sounds; a 100–400 Hz bandpass filter from 89 to 116 s for voices; and a 100–600 Hz bandpass filter from 116 s to the end for the deep breathing, snoring, coughing, and swallowing motions. It is observed that this volunteer has about 9 breaths per minute from the respiration wave results and 90 pulses per minute from the pulse wave results. There is no recorded breath during the breath holding period and the intensity of breaths increases during the deep breathing period as expected. Recorded magnitudes do change during different physiological activities and the coughing action generates the largest magnitude in the respiration waves. The pulse waves are generally consistent throughout the experimental period with occasional larger magnitudes during different actions as those motions can increase the movements of neck tissues. The results of the audio wave in the time domain are further analyzed in the frequency domain in Figure 1e to show patterns for talking at around 120 Hz, heartbeats between 20 and 200 Hz, and breathing, snoring, swallowing patterns from 100 to 600 Hz. In another test on the neck region, experimental data collected before and after exercises are compared in Figure S23, Supporting Information. Results show the amplitude and frequency of respiration, pulse and heart sound waves increase after exercise and gradually reduce during the recovery state. The heart can respond to the body needs with increased blood ejection and oxygen consumption during exercise by adjusting the intensity (cardiac contractile reserve) and frequency (heart rate reserve) of myocardial contractions. Previous works have shown that the first heart sound is closely related to the cardiac contractility ^[^ ^35^ ^]^ and our results show the amplitude of the first heart sound (S1) changes significantly from 2.8 mV to 11 mV before and after the exercise while the second heart sound (S2) is about the same (Figure S24, Supporting Information). Exercise also results in an increase of the heart rate of the volunteer from 90 to 130 bpm, which implies that the cardiac regulation during the workout depends more on the cardiac contractile reserve rather than the heart rate reserve.

### 2.2 The Heart Sound and Korotkoff Sound

**Figure 2:**
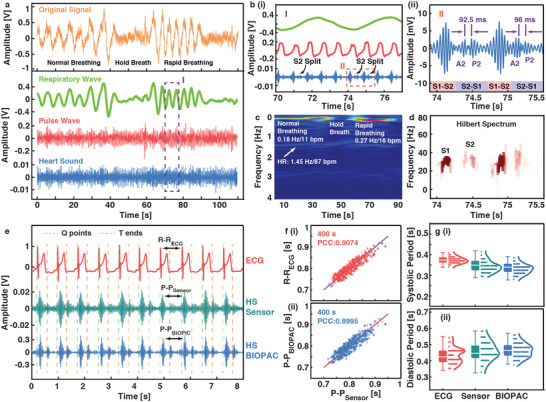
Measurement and analysis of heart sounds by placing the sensor patch at the heart location. a) Original signal and filtered signals acquired at the heart location. The respiratory wave is the baseline of the original signal without high frequency noises; the pulse wave and heart sound are obtained by using the 10 Hz low‐pass filter and the 20–200 Hz band‐pass filter after removing the baseline drift, respectively. b‐i) Enlarged view of the dotted box in a, showing the S2 split during the inspiration process. ii) Enlarged view of the dotted box in i), highlighting the aortic (A2) and pulmonic (P2) components during the S2 split. c) The STFT spectrum of the original signal (Figure 2a) within 0–4 Hz, showing the fluctuations of heart rate (HR) and respiration rate (RR). d) The Hilbert spectrum corresponding to the heart sound in b (ii). e) The comparison of the recorded heart sounds from the piezoelectret sensor (HS Sensor) and a medical physiological recorder (HS BIOPAC). The timing of the cardiac cycles is verified by the ECG reference. f) Comparison of the instantaneous cardiac cycles between the piezoelectret sensor (P‐P_Sensor_) and i) ECG (R‐R_ECG_), and ii) BIOPAC (P‐P_BIOPAC_). g) Calculation of the i) systolic period and ii) diastolic period length based on the data recorded by the ECG, piezoelectret sensor, and BIOPAC.

In the second experiment, heat sounds are further characterized by placing the sensor patch close to the heart position. Heart sound (HS) is an important mechano‐acoustic parameter which has been widely utilized in application such as disease diagnosis and emotion recognition. ^[^ ^16^, ^36^, ^37^ ^]^ In a normal heartbeat cycle, two sound components, S1 and S2, are observed due to the closing of the atrioventricular valves and the closing of the semilunar valves, respectively. **Figure** **2**a is the original recorded signal from the heart location with normal breathing, hold breath, and rapid breathing conditions as well as filtered physiological signals of different frequency ranges to reveal the respiratory wave, pulse wave, and heart sound. Figure 2b‐i shows the enlarged view from 70 to 77 s for the respiration, pulse, and heart sound results. The physiological splitting of S2 during the inspiration process can be observed in the enlarged view containing two parts, the aortic component (A2) and the pulmonic component (P2), which are related to the closure of the aortic valve and pulmonary valve, respectively. Inspiration increases the blood returning to the right‐heart and decreases the blood returning to the left‐heart, which leads to the extended systole of the right heart and the delayed closure of the pulmonary valve.^[^ ^38^ ^]^ The delay between A2 and P2 is found to be around 90 ms in the rapid deep breathing motion (Figure 2b‐ii). In the frequency domain, the short‐time Fourier transform (STFT) result of the original signal below 4 Hz is plotted in Figure 2c. It is found that the intensities of the respiration wave at 0.18 and 0.27 Hz are significantly stronger than those of the pulse waves at 1.45 Hz, and the three different breathing patterns (normal, hold and rapid) are clearly distinguishable. In the high frequency region, the Hilbert spectrum is used to analyze the instantaneous frequency of heart sounds in view of the small‐time difference between A2 and P2. The Hilbert–Huang transform can calculate the instantaneous amplitude/frequency of a signal with higher time‐frequency resolution than that of STFT^[^ ^39^ ^]^ as shown in Figure 2d, where the S2 split is clearly found in the Hilbert spectrum and the frequency of S2 is slightly higher than that of S1.^[^ ^40^ ^]^ Sounds generated by heart beats have also been detected on other locations, such as the carotid artery and brachial artery (Videos S1–S3, Supporting Information) with corresponding time‐frequency domain results summarized in Figures S25–S27 (Supporting Information).

A cycling test has been conducted to verify the stability and reliability of the piezoelectret sensor for the long‐term monitoring of heart sounds. The piezoelectret sensor (HS Sensor) and a medical‐grade physiological recorder (HS BIOPAC) are used to record heart sounds for 400 s simultaneously with the synchronized ECG signal as the reference (Figure 2e). First, the instantaneous cardiac cycles are calculated based on the R peaks of ECG signals (R‐R_ECG_) and the S1 peaks of heart sounds (P‐P_Sensor_, P‐P_BIOPAC_). It is found that the cardiac cycle results from the piezoelectret sensor are consistent with those from ECG and BIOPAC, with high Pearson correlation coefficient (PCC) values of 0.9074 and 0.8995, respectively in Figure 2f‐i,ii. Moreover, the length of the systolic period and diastolic period can be estimated and compared. The QRS complex in ECG represents the depolarization of ventricles and the beginning of cardiac systole, while the T wave corresponds to the physiological activities of ventricular repolarization and diastole.^[^ ^41^ ^]^ Therefore, S1 follows the Q‐point immediately and S2 occurs near the T‐end (Figure 2e), since S1 and S2 are generally considered to be related to cardiac systole and diastole, respectively. The systolic and diastolic lengths are expressed by Q‐T intervals and T‐Q intervals in the ECG, whereas the heart sound signal is divided into the S1 and S2 segment to calculate these two durations (Figure S28, Supporting Information). Similar results from the three signals are plotted in Figure 2g‐i,ii, which validates that the piezoelectret sensor can detect heart sounds accurately. Further analysis revealed that a high consistency has been achieved between the cardiac cycle and diastolic period with PCC (Pearson correlation coefficient) of 0.9404 rather than the systolic period with PCC of 0.1415 for a volunteer at the resting state, indicating that the fluctuation of the heart rate is mainly caused by the fluctuation of the diastolic period (Figure S29, Supporting Information).

**Figure 3:**
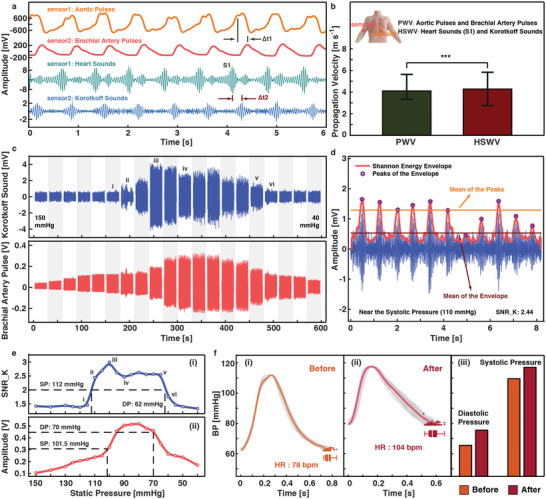
Measurements of Korotkoff sounds by placing the sensor patch at the upper arm brachial artery location for blood pressure estimation applications. a) Simultaneous measurements of the aorta (sensor 1) and brachial artery (sensor 2). The pulse wave velocity (PWV) is calculated based on aortic pulses and brachial artery pulses while the heart sound wave velocity (HSWV) is obtained according to heart sounds (S1 is used in this work) and Korotkoff sounds. b) Consistency between PWV and HSWV, with an intraclass correlation coefficient of 0.789 (***p < 0.001). Error bars correspond to the maximum and minimum values of the propagation velocity among the pressures from 75 to 105 mmHg, with corresponding center values shown. c) Recorded Korotkoff sounds and brachial artery pulses under corresponding static pressures. d) The analysis of the Korotkoff sounds such as the Shannon energy envelope and the calculation of the parameter SNR_K. e) BP estimation using the i) Korotkoff sound method, and ii) oscillometric method. f) Effects of caffeine stimulus on BP and HR. BP/HR measurement results by the Korotkoff sound method i) before and ii) after the intake of the caffeine drink, and iii) validation from the Omron system.

In the third experiment, the focus is the heart sounds detected on the brachial artery, or the “Korotkoff sounds.” Prior studies have attributed Korotkoff sounds to the blood turbulence and vascular wall instability while a unified view has not been established. Here, physiological signals on the aorta (aortic pulses and heart sounds) and the brachial artery (brachial artery pulses and Korotkoff sounds) are collected simultaneously (Figure S30, Supporting Information), with a measured distance of 0.82 m between the two locations in our experiment. The pulse wave velocity (PWV) is calculated based on the time delay between the aortic pulses and brachial artery pulses (Δt1). The heart sound wave velocity (HSWV) is calculated by the time difference of heart sounds (S1 is used in this calculation) and Korotkoff sounds (Δt2) in **Figure** **3**a. Korotkoff sounds can only be generated under specific static pressures and the pressure level of 75–105 mmHg is applied on the brachial artery using an inflatable bladder attached to a mercury meter. The average propagation velocity of 4.2 and 4.3 m s^−1^ is calculated for PWV and HSWV, respectively with an intraclass correlation coefficient of 0.789 (*p* < 0.001, Figure 3b), which is consistent with previously published values.^[^ ^38^, ^42^, ^43^ ^]^ The propagation velocities of PWV and HSWV under different static pressures are plotted in Figure S31 (Supporting Information) with a similar trend. These results imply that Korotkoff sounds and heart sounds (S1) share the temporal consistency indicating they propagate sequentially to cause vibrations at the body surface. The general understanding is that Korotkoff sounds are also generated from the blood ejection and turbulence process to the blood vessel as the origin of S1 is the cardiac ejection and closure of the atrioventricular valve induced by the ventricular systole.

The Korotkoff sound method is the gold standard of noninvasive blood pressure (BP) measurements in the medical community.^[^ ^44^ ^]^ In general, there will be no audible sound when a pressure above the systolic pressure is applied on the brachial artery by the cuff since the blood flow has been completely occluded. Subsequently, five phases of the Korotkoff sounds appear sequentially as the cuff pressure decreases: the systolic pressure when the first loud Korotkoff sound appears (phase I); a slight muffling and soft pulsating sound (phase II); the increase of the sound volume (phase III); the abrupt fall of the sound intensity (phase IV); and the diastolic pressure for the last audible sound (phase V). However, the appearance/disappearance of Korotkoff sounds is difficult to be characterized as it largely depends on the experience of the doctor and the white coat effect of some patients can also lead to wrong BP results when doctors are around.

An accurate wearable electronic stethoscope could help frequent BP detections without doctors. Here, the piezoelectret sensor is used to record the mechanical vibrations over the antecubital fossa, and a parameter, SNR_K, is proposed to characterize Korotkoff sounds for blood pressure measurements. As the static pressure applied by the bladder cuff attached to a mercury manometer gradually decreases from 150 to 40 mmHg (Figure S32, Supporting Information), both Korotkoff sounds and brachial artery pulses are measured simultaneously for each 30‐s static pressure (Figure 3c). Korotkoff sounds under 110 mmHg (close to the systolic pressure) are drawn in Figure 3d and analyzed as an example. Results from other pressure levels are shown in Figure S33 (Supporting Information). It is difficult to identify the Korotkoff sounds manually due to the poor signal‐to‐noise ratio around the systolic/diastolic pressure. In this work, a parameter, SNR_K, is proposed as the ratio of the mean of Shannon energy envelope peaks to the mean of overall Shannon energy envelope by using the 2‐order Shannon energy envelope as the estimation

(1)

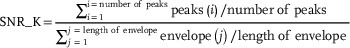




Experimentally, a threshold of 2 is chosen to define the appearance/disappearance of Korotkoff sounds since it means that the Korotkoff sounds have an approximate intensity level similar to that of the background noises and can barely be distinguishable. When the cuff pressure is near the systolic pressure, the amplitude of Korotkoff sounds might fluctuate greatly since the blood flow is in the critical state of complete blockage and partial ejection (Figure S33a,b, Supporting Information). On the other hand, the SNR of Korotkoff sounds reduces rapidly as the blood flow becomes less turbulent near the diastolic pressure (Figure S33e,f, Supporting Information). The Korotkoff sounds appear obviously between these two pressures and can be clearly distinguished by SNR_K (Figure S33c,d, Supporting Information). Overall, the SNR_K and a threshold of 2 are found to be adequate for these complex situations to accurately identify the systolic and diastolic pressures, as shown in Figure 3e‐i. The estimated BP results are 112/62 mmHg, which are close to those from the reference BP monitor (insets of Figure S32 in the Supporting Information with the BP reference result of 109.5/65.5 mmHg). The traditional oscillometric method can also evaluate BP values by measuring the brachial artery pulses concurrently. In this case, a prior calibration process based on different users is necessary similar to those used in the commercial BP monitors. When using the fixed ratio (0.55 for the systolic pressure, 0.85 for the diastolic pressure) from the literature, ^[^ ^45^, ^46^ ^]^ large systematic errors of BP measurements are found as shown in Figure 3e‐ii.

Compared with the blood pressure estimation models based on pulse wave transit time and machine learning algorithms,^[^ ^17^, ^47^ ^]^ the Korotkoff sound method based on the SNR_K parameter takes the advantages of reliable accuracy and prior dataset‐free and is easier to be transplanted into wearable medical devices. Blood pressure measurement results from seven volunteers are shown in **Table** **1** by using the prototype sensor and a medically certified Omron system. In this case, volunteer #7 has the condition of slight hypotension, which has been diagnosed by both methods. In a second test, BP measurements are performed by the Korotkoff sounds obtained at the radial artery for two volunteers (#8, #9) as summarized in Table S1 and Figure S35 (Supporting Information). The weak sound intensity at the radial artery (Figure S36, Supporting Information) results in slightly higher errors (within ± 10 mmHg). The capability of using the Korotkoff sound method to track the fluctuation of the dynamic BP is also demonstrated by taking BP measurements for a volunteer before and after consuming caffeine‐based drink. Caffeine is a common chemical stimulus to affect BP and heart rate (HR) by regulating the release of stress hormones. For the volunteer without the caffeine habit (#6), BP and HR are evaluated before and 30 minutes after the consumption of the caffeine drink. Significant increases in both physiological parameters (112/62 mmHg → 117.5/80.5 mmHg for BP and 78 bpm → 104 bpm for HR) have been observed after the caffeine intake (Figures 3f‐i,ii), which is consistent with results disclosed in previous works.^[^ ^48^ ^]^ In this test, the Omron system has also been used as the reference for simultaneous detections with results of 109.5/65.5 mmHg → 117/75.5 mmHg for BP and 77.5 bpm → 99.5 bpm for HR (Figure 3f‐iii; Figure S37, Supporting Information).

**Table 1:** Comparison of the BP results measured by the commercial blood pressure monitor (Omron BP7211) and the Korotkoff sound method

SBP/DBP [mmHg]^a)^	#1	#2	#3	#4	#5	#6	#7
Omron^b)^	109/64.5	110/71.5	111/76	123.5/78.5	102.5/65	109.5/65.5	105/57
Korotkoff sound	113/62.5	107/72.5	108/71.5	127/77.5	97.5/62	112/62	105/54.5
Differences	4/−2	−3/1	−3/−4.5	3.5/−1	−5/−3	2.5/−3.5	0/−2.5

^a)^ The detailed results of the two methods are shown in Figure S34 (Supporting Information);

^b)^ BP measurement is performed twice for each volunteer using the Omron monitor and the mean is taken as the BP reference result.

### 2.3 Breath Sound

**Figure 4:**
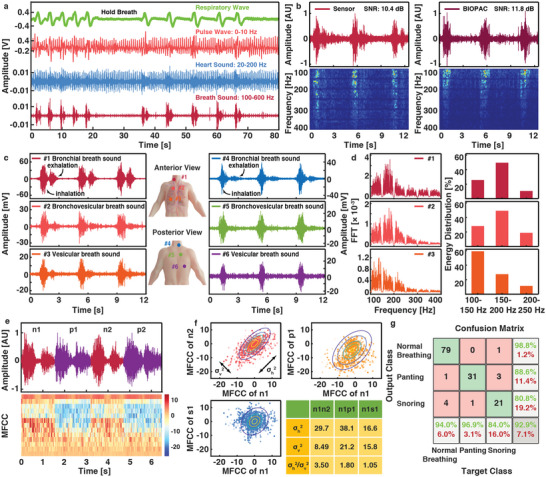
Acquisition and classification of breath sounds. a) The typical sound record from the left chest location during the deep breath is separated to various physiological signals of different frequency bands, including respiratory wave, pulse wave, heart sound, and breath sound. b) Comparison of the breath sounds and STFT spectrum obtained from: a piezoelectret sensor, and a medical recorder BIOPAC. c) Time‐series morphologies, and d) frequency components of the bronchial, bronchovesicular, and vesicular sounds. The original breath sound data is processed by a digital comb filter to suppress the power frequency (50 Hz) component and the high‐order harmonic components. e) Similar breath patterns (n1, n2 for normal breathing and p1, p2 for panting) in the magnitude versus time plot (top) can be distinguished in the MFCCs plot qualitatively (bottom). f) Quantitative analyses by using a parameter (*σ*_h_^2^/*σ*_v_^2^) to quantify the MFCCs similarity between two breath sounds, respectively. g) Confusion matrix of the classification results for three breathing patterns (normal breathing, panting, and snoring).

In the fourth experiment, breath sounds are detected and analyzed. In practice, doctor often uses a stethoscope to check breath sounds by asking the patient to breathe deeply to examine the crucial aspects of the respiratory system and diagnose respiratory diseases (such as asthma). In this work, a piezoelectret sensor is placed on the left chest location to examine the physiological information of deep breathing conditions for the respiratory wave, pulse wave, heart sound, and breath sound as shown in **Figure** **4**a. It is found that the prototype sensor has achieved a SNR comparable to those of a medical recorder BIOPAC (10.4 dB vs 11.8 dB) and the corresponding STFT results suggest that the energy of normal breathing is mainly concentrated between 100 and 400 Hz (Figure 4b).^[^ ^49^ ^]^ The frequency bands of heart sounds (especially S2) and breath sounds can partially overlap to interfere results with each other (Figure S38, Supporting Information). Researchers have tried to separate heart sounds and breath sounds by using complex algorithms (such as the independent component analysis, ICA).^[^ ^50^ ^]^ To reduce the interference of heart sounds, the breath sounds in following experiments are collected from the right side of the human body for reduced heart sounds. Generally, breath sounds are clearly audible in several positions along the trachea and lung (such as the neck, Figure S39, Supporting Information). In this section, three special positions are selected for breath sounds in the front and back of a volunteer for morphology and frequency characterizations (bronchial sounds of #1, #4; bronchovesicular sounds of #2, #5; and vesicular sounds of #3, #6) with results shown in Figure 4c. Bronchial sounds appear as air moves through the trachea and they have the strongest intensity and highest pitch in both inspiration and exhalation processes to be heard best over the trachea on the anterior and posterior portion of the neck. Bronchovesicular sounds occur when air moves through the large airways of the lungs with medium pitched sounds to be heard best over the 1st and 2nd intercostal space beside the sternum on the anterior side of the chest and between the shoulder blades on the posterior chest. Vesicular sounds are produced as air moves through the smaller airways in the lungs with the characteristics of low pitch to best heard over the entirety of the lung fields. Similar results can be found on another volunteer (Figure S40, Supporting Information). In the frequency domain, the frequency components of breath sounds gradually move to the low‐frequency region with the transmission of air flow along the respiratory system (Figure 4d), which is attributed to the absorption of the high frequency components when sounds pass through the lung.^[^ ^49^ ^]^

Accurate classification of breath sounds is vital for the diagnosis of respiratory diseases. In hospitals, trained doctors characterize the classification by their own experiences with the help of stethoscopes. However, most respiratory diseases may not happen all the time. A simplified and accurate classification method is required to meet the increasing needs for home diagnosis. Specifically, Mel‐frequency cepstral coefficients (MFCCs) have been successfully applied in speech recognitions ^[^ ^51^ ^]^ and proposed to classify breath sounds in this work (Note S2, Supporting Information) due to the similar time‐frequency characteristics between the breath sound and speech. MFCCs are coefficients that collectively make up the mel‐frequency cepstrum (MFC), which is the representation of the short‐term power spectrum of a sound by conducting a linear cosine transform of a log power spectrum on a nonlinear mel scale of frequency.^[^ ^51^ ^]^ In this work, no complex algorithms (such as hidden Markov model, artificial neural networks ^[^ ^52^ ^]^) are involved since the category and variation of breath sounds are far lower than those of speech, which greatly reduces the computational requirements. In the prototype demonstration, three kinds of breath sounds are used in the classification process: normal breath, panting after strenuous exercise, and snoring. From the MFCCs results in Figure 4e, it is found that breath sounds are similar in the amplitude versus time plots but vary greatly in the frequency versus time analyses by means of MFCCs. Specifically, results show visual periodic changes in the MFCCs plot from the time series of “normal breathing (n1) – panting (p1) – normal breathing (n2) – panting (p2)” as an example for the qualitative characterization. For quantitative analyses of the variations, a parameter, *σ*_h_^2^/*σ*_v_^2^, is introduced after mapping the MFCCs results of two breath sounds into a scatter plot, where *σ*_h_^2^ and *σ*_v_^2^ are the variances of results along the parallel and perpendicular direction to the diagonal axis (*y = x*), respectively (Figure 4f). MFCCs of the breath sound sample from the normal breathing (n1) are compared to those of breath sounds from all three categories (n2 for normal breathing, p1 for panting and s1 for snoring) respectively, and results indicate that breath sounds from the same category achieve the largest *σ*_h_^2^/*σ*_v_^2^ value. A large *σ*_h_^2^/*σ*_v_^2^ value indicates high similarity, while a small value means high dissimilarity since points from two sets of identical values would be mapped to the diagonal axis (*y = x*) to result in the *σ*_h_^2^/*σ*_v_^2^ value of infinite. Therefore, *σ*_h_^2^/*σ*_v_^2^ results between each collected breath sounds and the prepared templates corresponding to each breath patterns are calculated for the classification to the right category with the highest *σ*_h_^2^/*σ*_v_^2^ value (Figure S45, Supporting Information). The model realizes a high accuracy of 92.9% as shown in Figure 4g in this example with the F1 score of normal breath, panting and snoring of 0.9634, 0.9254, and 0.8235, respectively. Another commonly used method, dynamic time warping,^[^ ^51^ ^]^ is used to measure the similarity between two time series to quantify the similarity of MFCCs and achieves a classification accuracy of 87.2% (Figure S46, Supporting Information). In addition, different volunteers could also be recognized by this algorithm (accuracy: 84.3%) for the potential application of using breath sounds in the human identity recognition process (Figure S47, Supporting Information).

### 2.4 Heart Sounds Sensor Array

**Figure 5:**
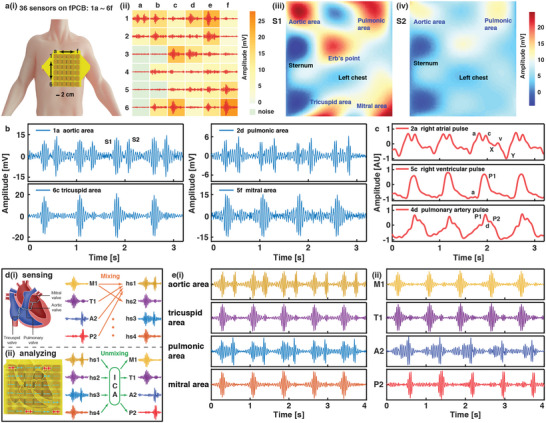
A 6×6 sensor array on the left chest to detect the heart sounds. a‐i) Mapping the heart sounds and pulses with the 6×6 sensor array. Scale bar: 2 cm. ii) Heart sounds measurement results simultaneously from 36 sensors. iii) S1, and iv) S2 intensity map from the sensing results by using the cubic spline interpolation scheme. b) Heart sounds from the Aortic area (1a), Pulmonic area (2d), Tricuspid area (6c) and Mitral area (5f). c) Morphological variability of the pulses at different locations, exhibiting pressure waveforms in the right atrium (2a), right ventricle (5c), and pulmonary artery (4d). d) Illustration of the four heart valve components and: i) mixing of heart sounds as the detected signals; ii) unmixing of detected signals by the ICA (independent component analysis) algorithm. e) Demonstration of heart sounds separation by the ICA. i) Original heart sounds from the four valve areas. ii) Corresponding four valve components after applying the ICA separation algorithm.

In the fifth experiment, a sensor array is used to characterize heart sounds of a large area around the chest simultaneously. This is like the practice for doctors to change auscultation positions in order to determine the location of lesion because the audible heart sounds are the mixture of different valve closures. A 6×6 sensor array (a single sensor unit of 2×2 cm^2^, spacing of 1 cm, and whole array of 17 × 17 cm^2^) is fabricated to examine the location dependency of amplitude and morphology of the heart sounds in **Figure** **5**a‐i. The sensor array has low crosstalk below −20 dB with the help of independent mechanical structures and good consistency with output amplitudes within ± 10% of the average value under the same mechanical stimulus (Figures S48 and S49 Supporting Information). Mechanical vibrations on the body surface are mapped by the sensing matrix, where intensified colors indicate stronger heart sounds in Figure 5a‐ii. Results show heart sounds can be detected in almost all units, while some units have weak sound signals due to the obstruction and absorption of the sternum, costal bones, and fat near the chest. The amplitudes of S1 and S2 are extracted for each sensor location and the mapping result after interpolations is exhibited in Figure 5a‐iii,iv, respectively. Five traditional auscultation areas of Aortic, Pulmonic, Erb's point, Tricuspid and Mitral regions are distinguished in the intensity map of S1, while the S2 map appears to have weaker signals. In general, S1 is audible in all auscultation areas because of the stronger loudness, while S2 has a weaker loudness and could only be perceived near the semilunar valves.^[^ ^17^ ^]^ The 36‐positions of 6×6 sensor array from the same volunteer is further examined using a medical physiological recorder BIOPAC and results show similar characteristics (Figure S50, Supporting Information). Detailed heart sounds from the four valves areas further illustrate the amplitude differences between S1 and S2 (Figure 5b). Pulses in various shapes from 36 positions are acquired concurrently to exhibit comprehensive cardiac information (Figure S51, Supporting Information). For example, the pulse waveforms reflecting the pressure of the right atrium, right ventricle and pulmonary artery are captured by the sensor units above the correlated positions, similar to the possible implanted pressure catheters in hospitals (Figure 5c). Pulses from unit 2a have the typical biphasic waveform and the five identifiable components (a, c, v, X, Y) corresponding to the physiological activities of right atrial contraction/relaxation, tricuspid valve closing/opening, respectively.^[^ ^29^ ^]^ The right ventricular pulses (from unit 5c) contain a small “a” wave, representing right atrial systole, and a swift upstroke (P1 wave) representing right ventricular systole.^[^ ^53^ ^]^ In the pulmonary artery pulses (from unit 4d), the dicrotic peak (P2) and dicrotic notch (d) are observed due to the closure of the pulmonary valve.^[^ ^53^ ^]^

In general, S1 and S2 are composed of two components corresponding to the closure of mitral valve (M1), tricuspid valve (T1), aortic valve (A2), and pulmonary valve (P2), respectively and all audible heart sounds are the mixture of these four valve components for a healthy volunteer (Figure 5d‐i). It is important to separate the four independent components from the mixed heart sounds for doctors to accurately analyze patient's symptoms with specific lesions (such as mitral stenosis, aortic valve insufficiency, etc.), rather than changing the auscultation areas frequently to compare the differences in volume and frequency at different positions. This work separates these components using the data from the sensor array and the independent component analysis (ICA) algorithm (Figure 5d‐ii). ICA is a separation algorithm which has been widely used in the separation of physiological signals. ^[^ ^50^ ^]^ The heart sounds data with the best SNR are selected from each of the four valves areas (Figure 5e‐i), and the separation results processed by ICA are shown in Figure 5e‐ii. The time differences between M1 and T1, A2 and P2 during normal breaths are about 31 and 22 ms, which is consistent with values found in the previous literature. ^[^ ^38^, ^54^ ^]^ Furthermore, the ICA algorithm is successfully used on the heart sounds data collected from another volunteer, with the time difference of about 35 and 27 ms (Figure S52, Supporting Information). These separated heart sounds components reflect the instantaneous behaviors of four heart valves, which can potentially provide accurate diagnoses for indistinguishable heart diseases.

## 3 Conclusion

Audio physiological signals, including heart sounds, Korotkoff sounds, and breath sounds, provide important physiological information. However, poor signal‐to‐noise ratio, weak intensity level, and mixed frequency responses are often challenging factors to block the detection of high‐quality audio physiological data by flexible electronic devices. This paper introduces the combination of innovative hardware designs and advanced software analyses to demonstrate the feasibility of reliably monitoring heart sounds, Korotkoff sounds, and breath sounds. In particular, high‐fidelity physiological signals is recorded using the folded double‐layer piezoelectret sensor and sensor array with the shielding and protecting packaging layer for potential clinical applications in various scenarios. The outstanding piezoelectret sensors can accurately acquire subtle vibration signals for the real‐time monitoring of a variety of physiological activities, such as respiratory waves, pulse waves, heart sounds, breath sounds, snoring, talking, coughing and swallowing. Experimental results show detected signals are well matched with the current state‐of‐art medical recorder BIOPAC for heart sounds and the successfully extracted ventricular systolic and diastolic periods are consistent with ECG references. The 6×6 sensor array placed on the left chest can map the morphology and amplitude distributions of heart sounds, as well as the associated pulses. Furthermore, heart sound components separated by the ICA algorithm correspond well to the instantaneous mechano‐acoustic behavior of four heart valves as a new approach for heart diseases diagnoses. In another application, Korotkoff sounds and the proposed resulting parameter, SNR_K, are capable of BP measurements without the involvement of real doctors and prior calibration. As a practical demonstration example, BP increases caused by the intake of caffeine stimulation are captured by the Korotkoff sound measurements and characterizations. A range of analyses for breath sounds, including the comparison of the morphology and frequency at different positions, and the classification of breath sounds from different patterns and volunteers, indicate their potential applications in family diagnosis and identity recognition. While clinical validations involving more participants will be of interest in the future research, the current results exhibit key proof‐of‐concept validations toward self‐monitoring of cardiovascular and respiratory devices for next generation systems.

## 4 Experimental Section

### Fabrication of the Folded Double‐Layer Piezoelectret Sensor

Detailed preparation of the bonded double‐layer FEP film with crisscross cavities can be found in the previous work.^[^ ^29^ ^]^ After adhering a copper tape (external electrode, with a thickness of 20 µm and an area of 4×2 cm^2^) on one side of the FEP film, a negative power supply (DW‐N303‐1ACH2, Dongwen High Voltage Power Supply (TianJin) Co., Ltd) was used to perform the corona charging process. The FEP film (3 cm below the corona needle) was exposed to the electric field of −20 kV for 5 minutes when the external electrode was grounded. Subsequently, two identical copper tapes (slightly smaller than 2 × 2 cm^2^) were placed on the other side as the internal electrodes, and one of the copper tapes was glued with a polyimide tape (20 µm) to prevent short circuit between these two internal electrodes. Finally, the whole structure was folded in half along the central axis to encapsulate the internal electrodes inside. In this process, the shielding layer envelops the entire sensor since the external copper electrode is slightly larger than the internal FEP films to achieve sufficient electromagnetic shielding.

### Fabrication of the Heart Sounds Sensor Array

36 piezoelectret sensors were prepared in advance using the above method. These sensors were glued to a customized flexible printed circuit board (fPCB) with double‐sided tape to form the 6×6 array. A single sensor unit had an area of 2×2 cm^2^ and a spacing of 1 cm to the neighboring unit. The internal electrodes of each sensor were welded on the corresponding pads through copper wires, and the fPCB was connected with the subsequent circuits using the zero insertion force connectors. The leads among different sensor units were electrically insulated to reduce the possible crosstalk.

### Characterization of the Piezoelectret Sensor

The morphology of FEP grooves was examined by the surface profilometer (AlphaStep P‐7, KLA‐Tencor Corp.) and the optical microscope (OLYMPUS BX53MTRF‐S). The surface potential of FEP films and PET films was measured using an electrostatic voltmeter (Model 347, TREK INC.). The modal shaker (SA‐JZ002, Wuxi Shiao Technology Co., Ltd.) and force sensor (AT8601‐20N, Suzhou Autoda Automation Equipment Co., Ltd.) were used by applying pressure with controlled amplitude and frequency to the DUT (Figure S6, Supporting Information). The output of the DUT was acquired by a current preamplifier (SR570, Stanford Research Systems), and was further integrated as the transfer charge. All data were sampled by NI USB‐6009 and NI LabVIEW 2017.

### Human Experiments

For the heart sound experiments, both the piezoelectret sensor and a physiological recorder (MP36, SS17L, BIOPAC Systems, INC.) were used to record the heart sounds simultaneously. The ECG reference was acquired by the AD8232 module.

For the Korotkoff sound experiments, seven volunteers (#1–#7) participated in the BP assessment at the brachial artery position and two other volunteers (#8, #9) participated at the radial artery position. Volunteer #6 was also tested for the effects of caffeine stimulants. Furthermore, BP measurements were performed using the commercial monitor (Omron BP7211) before and after each Korotkoff sound detection, and the average of the two measurements was considered as the BP reference value.

For the breath sound study, signals from the piezoelectret sensor were compared with those from BIOPAC (MP36, SS17L). A volunteer (v#1) was asked to breathe in normal, panting, and snoring patterns respectively as the classification templates. Afterward, 84 cases of normal breathing, 32 cases of panting, and 25 cases of snoring were collected to demonstrate the classification process. The 84 normal breathing cases of volunteer v#1 were also used along with the normal breathing from two other volunteers (42 cases for v#2, 14 cases for v#3) to demonstrate potential applications in human identity recognition. All breath sounds were collected in the neck to avoid the influence of the acquisition location on frequency and intensity.

For all the human experiments, the output of the piezoelectret sensor (or sensor array) was processed by a customized circuit (Figure S10, Supporting Information) and sampled by the NI USB‐6255.

### Statistical Analysis

For all the human experiments, the output of the piezoelectret sensor (or sensor array) was processed by the customized circuit (Figure S10, Supporting Information) and sampled by the NI USB‐6255. About 500 cardiac cycles within 400 s were recorded simultaneously by the piezoelectret sensor, BIOPAC and the ECG module AD8232 to compare the consistency (Figure 2e). Instantaneous cardiac cycles were calculated based on R1R intervals for the ECG and S1–S1 intervals for the piezoelectret sensor and the mapping results were plotted in Figure 2f‐i. Then, Pearson correlation coefficient was calculated using instantaneous cardiac cycles from the piezoelectret sensor and ECG as two correlational variables. The similar processing was performed between the piezoelectret sensor and BIOPAC and the corresponding results were summarized in Figure 2f‐ii. Moreover, the length of the systolic period and diastolic period was extracted from the 400s data and drawn in the form of box‐whisker plot using the MATLAB, as shown in Figure 2g. The five horizontal lines from top to bottom of each box are the maximum value, the 25th percentile, the median, the 75th percentile, and the minimum value. Outliers, defined as values that are more than 1.5 times the interquartile range away from the maximum and minimum values, have been removed. Static pressure between 75 and 105 mmHg with an interval of 5 mmHg was applied on the brachial artery successively to illustrate the consistency between the PWV and HSWV. The corresponding results were summarized in Figure 3b and the error bars corresponded to the maximum and minimum values of the propagation velocity among these pressures, with corresponding center values shown. The intraclass correlation coefficient and p value were calculated using the IBM SPSS Statistics with the modal of two‐way random, the type of absolute agreement, and the confidence interval of 95%.

## Conflict of Interest

The authors declare no conflict of interest.

## Supporting information

Supporting Information

Supplemental Video 1

Supplemental Video 2

Supplemental Video 3

## Data Availability

The data that support the findings of this study are available from the corresponding author upon reasonable request.
