# Supplementary material for: Health Monitoring via Heart, Breath, and Korotkoff Sounds by Wearable Piezoelectret Patches
Source: Adv Sci (Weinh). 2023 Aug 21;10(28):2301180. doi: 10.1002/advs.202301180 (PMC10558643; doi:10.1002/advs.202301180)
Supplement: Supplementary file 1 — Supporting Information [file ADVS-10-2301180-s001.pdf]

ADVANCED SCIENCE
Open Access

Supporting Information

for *Adv. Sci.*, DOI 10.1002/advs.202301180

Health Monitoring via Heart, Breath, and Korotkoff Sounds by Wearable Piezoelectret Patches

Liuyang Han, Weijin Liang, Qisen Xie, JingJing Zhao, Ying Dong, Xiaohao Wang* and Liwei Lin**

Supporting Information

Health Monitoring via Heart, Breath and Korotkoff Sounds by Wearable Piezoelectret Patches

Liuyang Han, Weijin Liang, Qisen Xie, JingJing Zhao, Ying Dong, Xiaohao Wang*, Liwei Lin**

L. Han, W. Liang, Q. Xie, J. Zhao, Prof. Y. Dong, Prof. X. Wang
Tsinghua Shenzhen International Graduate School
Tsinghua University
Shenzhen 518055, China
E-mail: dongy@tsinghua.edu.cn

Prof. X. Wang, Prof. L. Lin
Tsinghua-Berkeley Shenzhen Institute
Tsinghua University
Shenzhen 518055, China
E-mail: wang.xiaohao@sz.tsinghua.edu.cn

Prof. L. Lin
Department of mechanical engineering
University of California, Berkeley
Berkeley, U.S.A
E-mail: lwlin@berkeley.edu

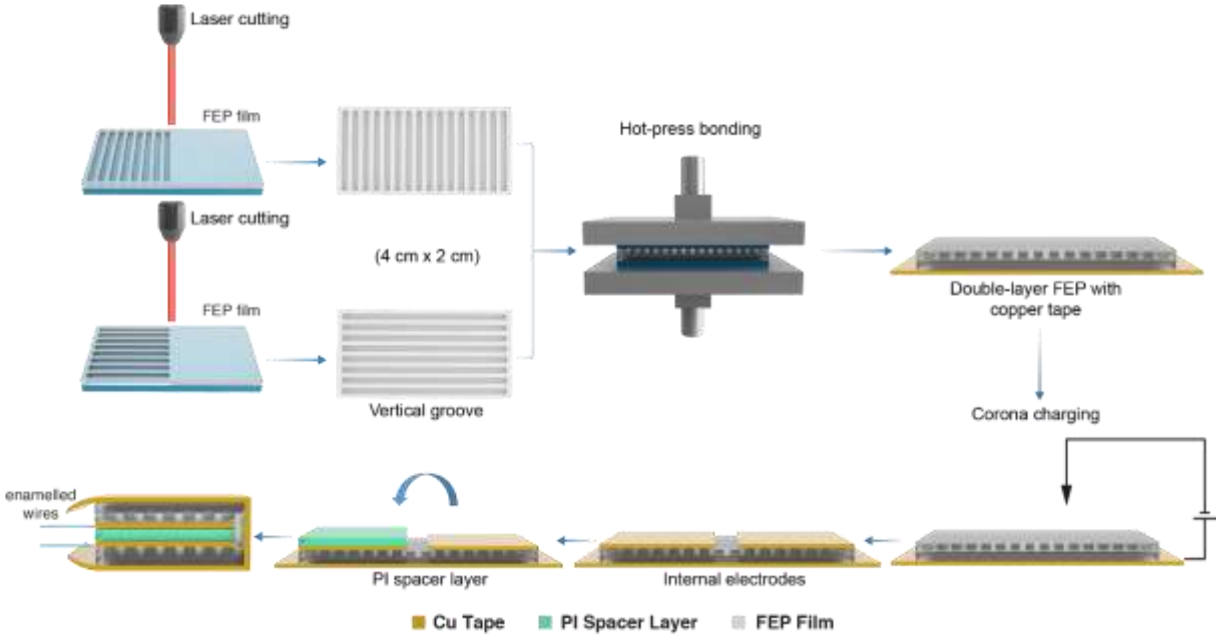

Figure S1. Fabrication process of the folded double-layer FEP piezoelectret sensor.

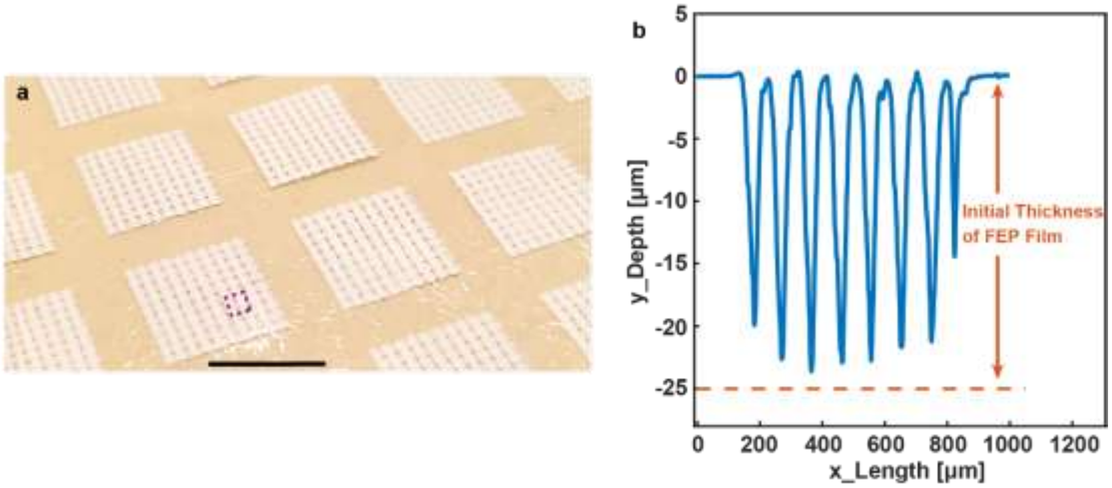

Figure S2. Characterization of the FEP piezoelectret sensor topography. (a) Photograph of the piezoelectret sensors, highlighting the crisscross grooves structure. Scale bar, 1 cm. (b) Topography characterization of a single-periodic groove in the dotted box in a using the surface profilometer. The FEP film (thickness of 25 μm) is grooved by a laser cutter, and the minimum thickness of the residual film is less than 2 μm.

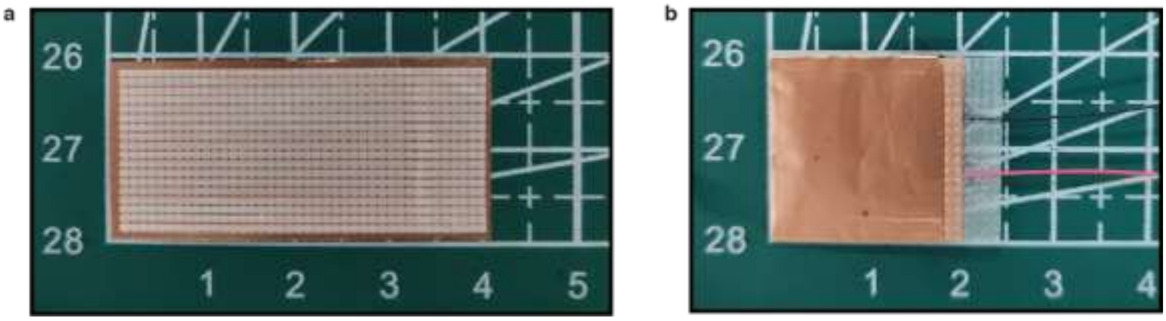

Figure S3. Photographic image of the piezoelectret sensor. (a) Double-layer FEP film placed on the shielding layer (copper tape). (b) The piezoelectret sensor after folding. Unit: cm.

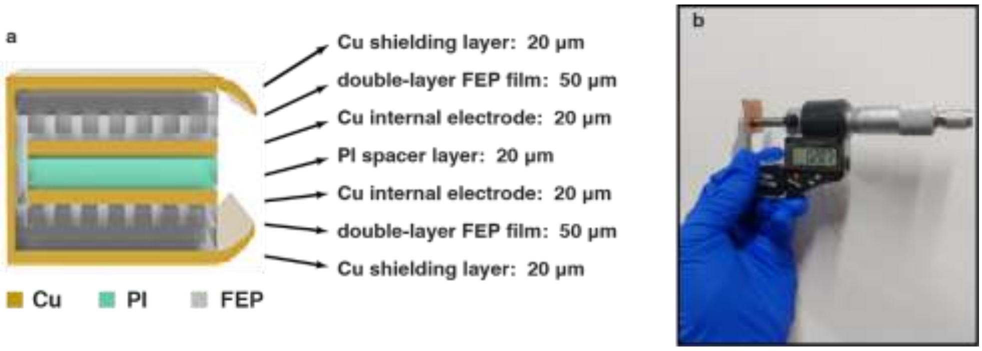

Figure S4. The thickness of the piezoelectret sensor. (a) Schematic diagram of the thickness of each layer in the sensor. The thickness dimension of the sensor has been greatly exaggerated. The shielding layer on the right side is opened to facilitate marking the thickness size of each layer. (b) The total thickness of the entire sensor.

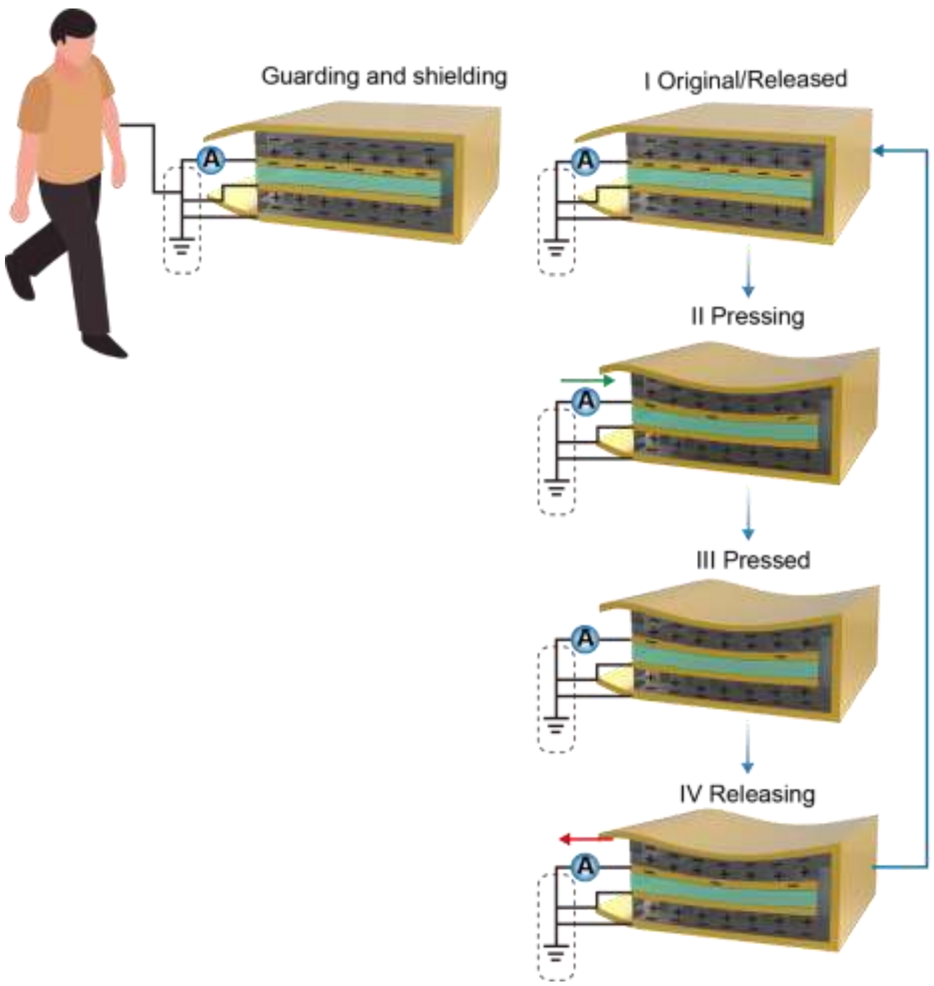

Figure S5. Working principle of the piezoelectret sensor. The thickness dimension of the sensor has been greatly exaggerated. The external electrode wrapping the entire sensor forms a grounded shield. The shielding layer on the left side is opened to highlight the charge distribution and electrical connection of each layer. The amount of inducted charges on the internal electrodes fluctuate periodically in response to the applied pressure cycles, generating alternating currents in the external circuit.

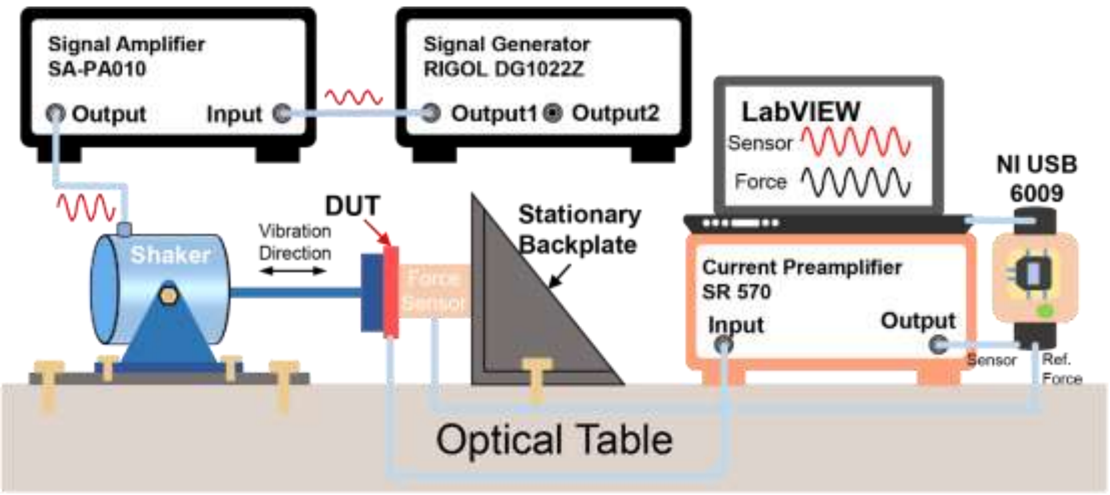

Figure S6. Experimental setup for characterizing the mechanical properties of the piezoelectret sensor. The modal shaker and force sensor mounted on an optical table are used apply pressure with controllable amplitude and frequency to the DUT. The output of the DUT is amplified by the current preamplifier and acquired by the NI USB 6009.

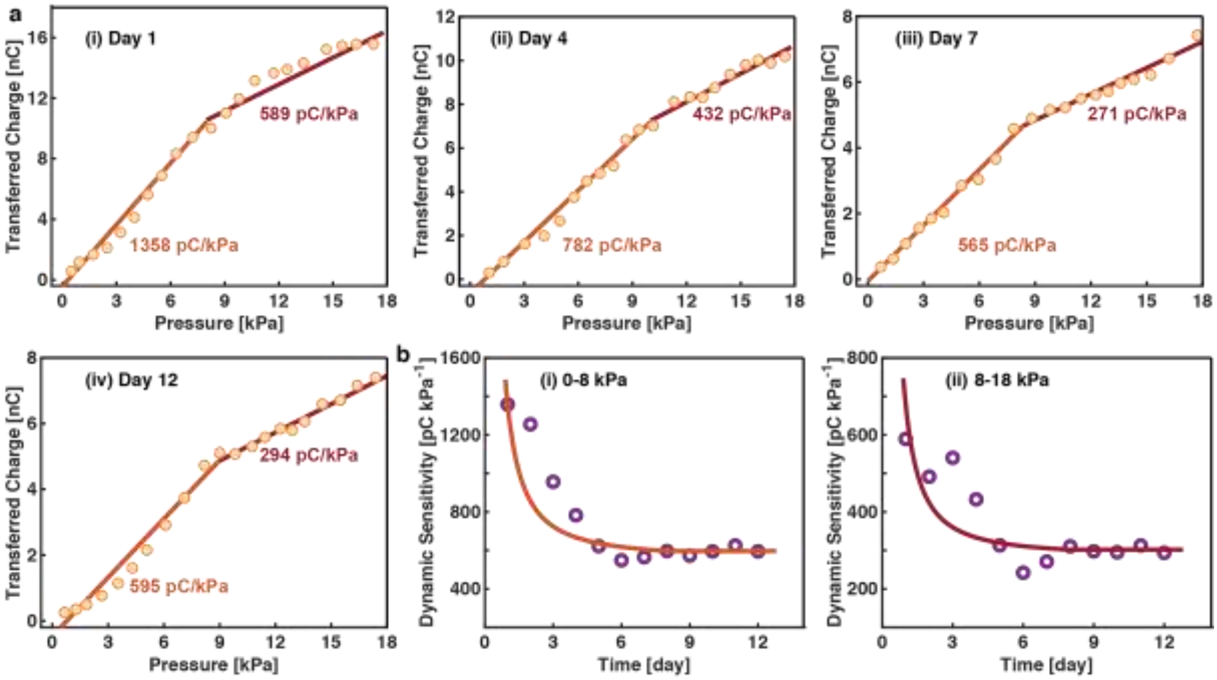

Figure S7. Variation of the dynamic sensitivity versus time of the piezoelectret sensor. (a) Results of transferred charges versus applied pressures at the specific date after the initial corona charging. (b) Results of dynamic sensitivity versus time. (i) Low-pressure region; (ii) High-pressure region. The sensitivity attenuation after the initial corona charging process is observed due to the charge neutralization process by the internal defects of FEP. After an initial attenuation of about one week, the dynamic sensitivity of the piezoelectret sensor eventually becomes stable.

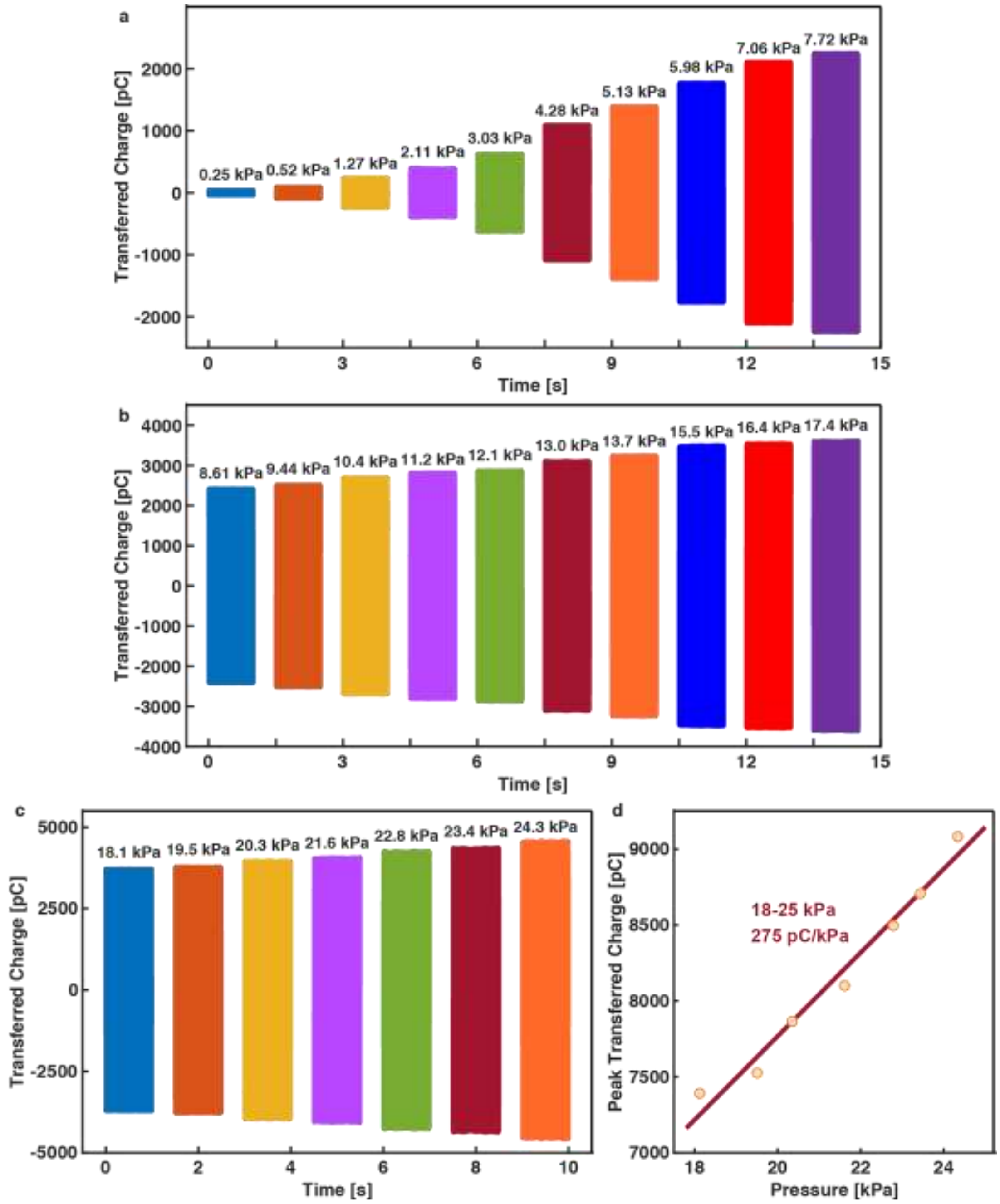

Figure S8. Transferred charges under different pressures. (a) Low-pressure region of 0-8 kPa. (b) High-pressure region of 8-18 kPa. (c) Transferred charges and (d) dynamic sensitivity of the same piezoelectret sensor in higher pressure regions (18-25 kPa). The dynamic sensitivity of this piezoelectret sensor in the range of 18-25 kPa is comparable to that of 8-18 kPa (275 pC kPa^{-1} v.s. 290 pC kPa^{-1}), indicating the potential to operate at higher pressures.

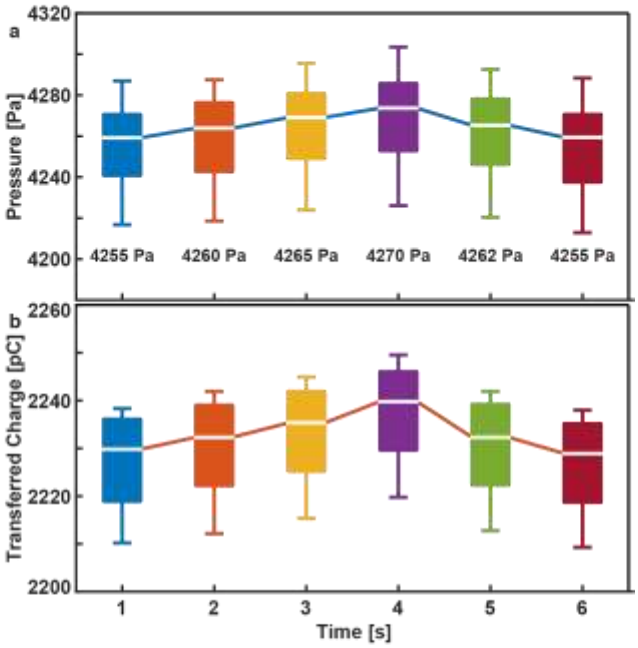

Figure S9. Pressure resolution test of the piezoelectret sensor. (a) Applied pressures. (b) Corresponding transferred charges. When the applied pressure is slightly changed around 4255 Pa (P0, which is the reference pressure chosen arbitrarily in the low linearity region), the corresponding transferred charges are measured. Although limited by the experimental setup, the applied pressure fluctuates within tens of Pascal, it can still be considered that a minute change in the applied pressure of 5 Pa causes a significant change in the transferred charge for the statistical results over a period of time.

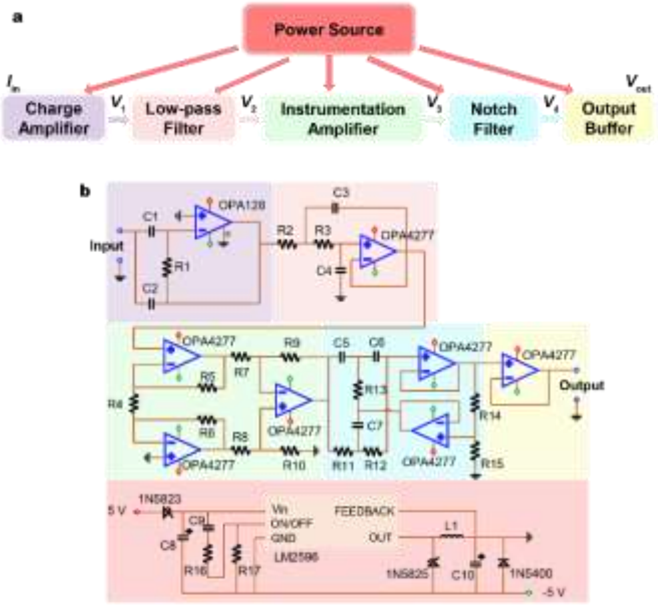

Figure S10. Scheme of the customized circuit. (a) Function modules of the circuit. (b) Detailed circuit diagram.

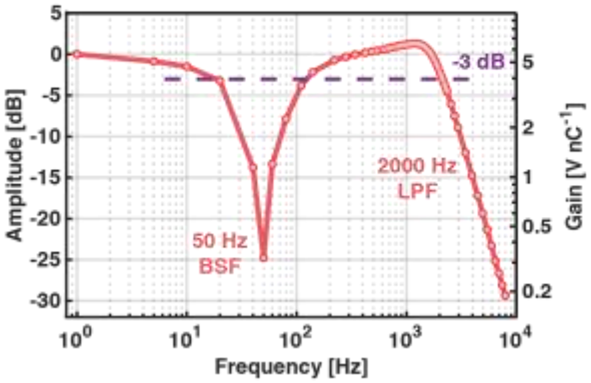

Figure S11. Amplitude-frequency response characterization of the customized circuit. This circuit contains a 50 Hz notch filter and a 2000 Hz low-pass filter with an amplitude gain of about 6 V nC^{-1}.

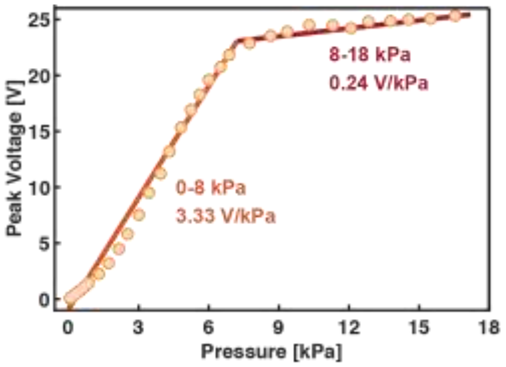

Figure S12. Dynamic sensitivity characterization using the customized circuit. A dynamic sensitivity property with two distinct linear regions are obtained (3.33 V kPa^{-1} for 0-8 kPa, 0.24 V kPa^{-1} for 8-18 kPa). A ±15 V supply is used to power the customized circuit, limiting the peak-to-peak value of the output voltage to within 30 V. The output voltage is close to the saturation threshold in the high-pressure region, resulting in a lower sensitivity.

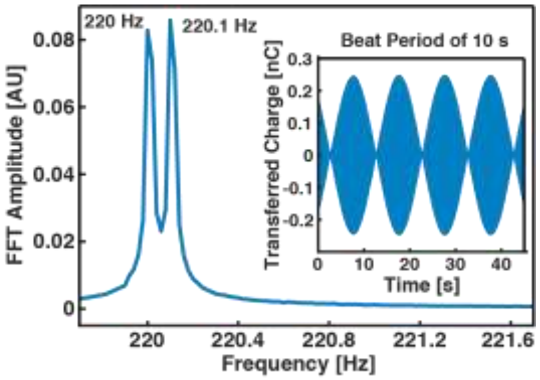

Figure S13. Pressure resolution characterization. Mixed pressures with a frequency difference of 0.1 Hz have been applied on the DUT and the time/frequency domain results of corresponding transferred charges are obtained.

Supplementary Note 1. Frequency response characterization of the piezoelectret sensor

Frequency response is a key parameter of dynamic sensing to acquire the resonant frequency and determine the operating range. Here, the frequency response of the prototype sensor is characterized using the experimental setup in Figure S4, with a varying frequency between 2-2000 Hz and a constant pressure amplitude of 1 kPa. Three different sensors with the same size (2 × 2 cm^2) and crisscross structure are involved in this characterization (Figure S14): FEP (piezoelectret) sensor with Corona charging (s1), FEP sensor without Corona charging (s2), and PET (non-piezoelectret) sensor without Corona charging (s3). Sensor s3 is the control group and the outputs mainly come from electronic noises and the triboelectric effect between the modal shaker and sensor electrodes (Figure S15). It is noted that the sensor s2 has a certain output intensity (rather than zero-output) without the Corona charging process, which could be attributed to the residual surface charges on the FEP film because of the friction between FEP and the outside during the fabrication process (Figure S16). Results of the sensor s1 show a first-order resonant frequency of about 740 Hz and a wide working range of 600 Hz as shown by the red line in Figure S14. The short-circuit current measurements from the three sensors with respect to frequency are presented in Figure S17. Furthermore, the frequency response is also characterized using the customized circuit, and similar results are obtained to show the first-order resonant frequency of about 820 Hz and the working range of 600 Hz (Figure S18).

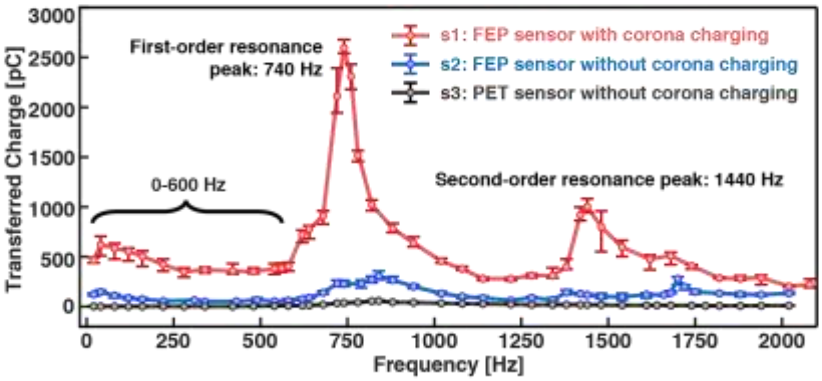

Figure S14. Frequency response characterization. The applied pressure has a variable frequency of 2-2000 Hz and a constant intensity of 1 kPa. Five identical measurements are performed for each frequency point with error bars showing the mean, maximum and minimum.

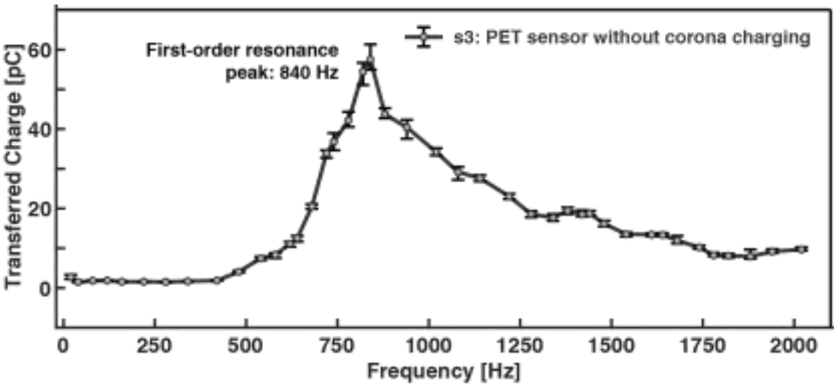

Figure S15. Enlarged view of the control group's amplitude-frequency response curve.

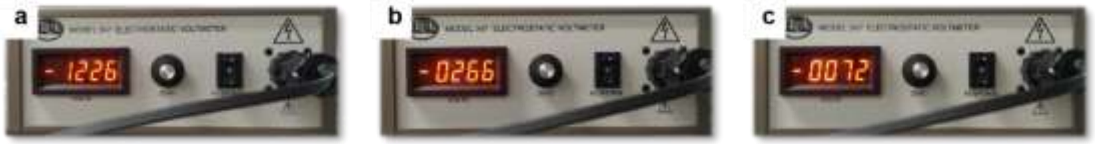

Figure S16. Surface potentials of the (a) FEP film with corona charging, (b) FEP film without corona charging and (c) PET film without corona charging.

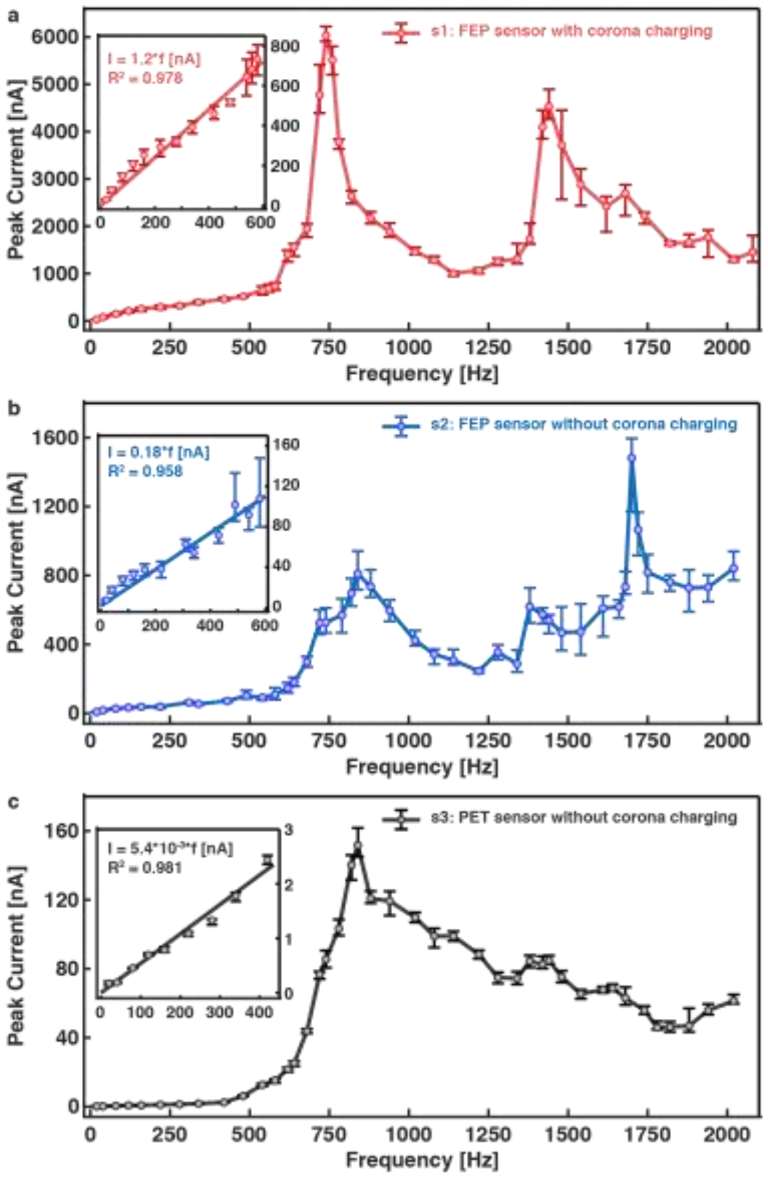

Figure S17. Output peak currents of the three sensors as the function of frequency. The amount of transferred charges is only related to the pressure amplitude, so the flat response results are observed in the working frequency band (0-600 Hz) of the transferred charge vs. frequency plot (Figure S11). However, the peak current depends on both the amplitude and frequency of the pressure, and the linear relationship is obtained in the peak current vs. frequency plot (0-600 Hz in the inset).

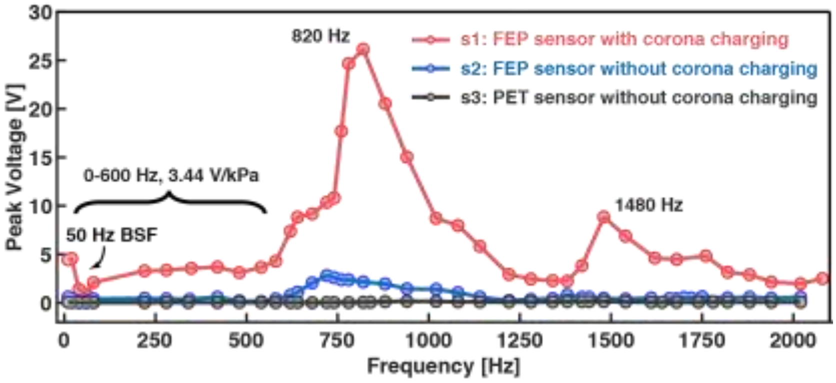

Figure S18. Amplitude-frequency response curves measured by the customized circuit. The similar linear working range (0-600 Hz) is obtained, with a gain of about 3.44 V kPa^{-1}.

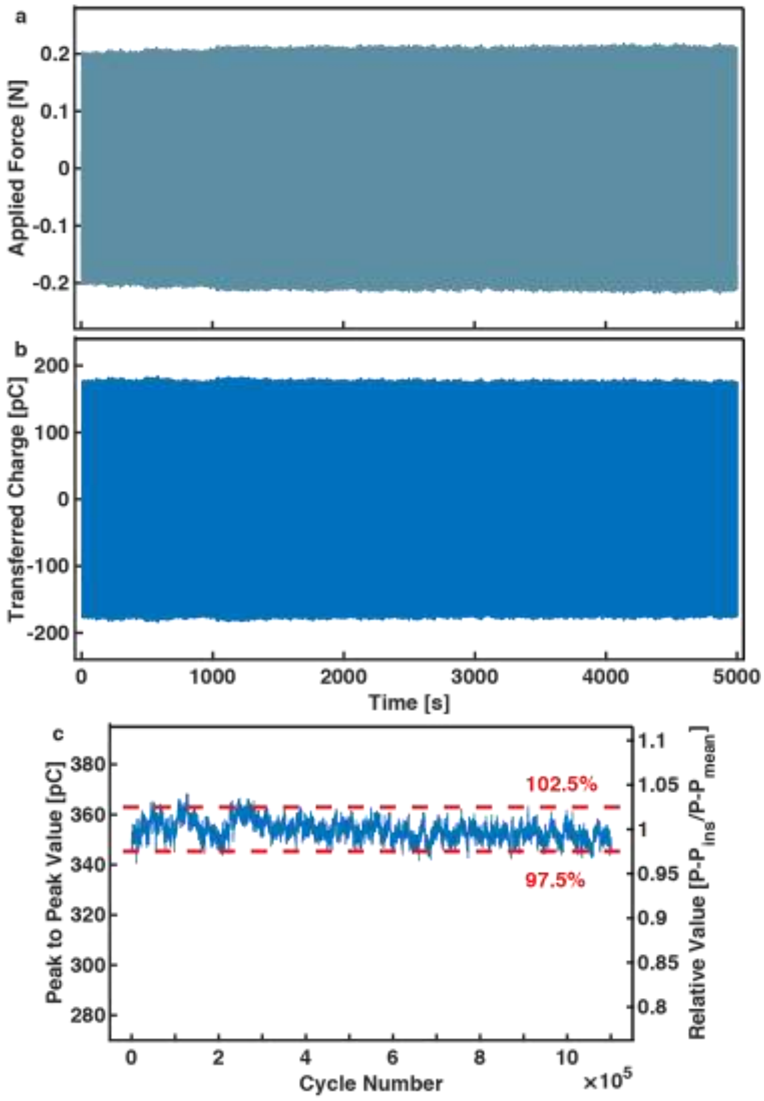

Figure S19. Mechanical stability test of the piezoelectret sensor. More than 1.1 million cycles of the (a) applied force and (b) corresponding transferred charges for 5000 s. (c) Fluctuation in the amount of transferred charges throughout the test.

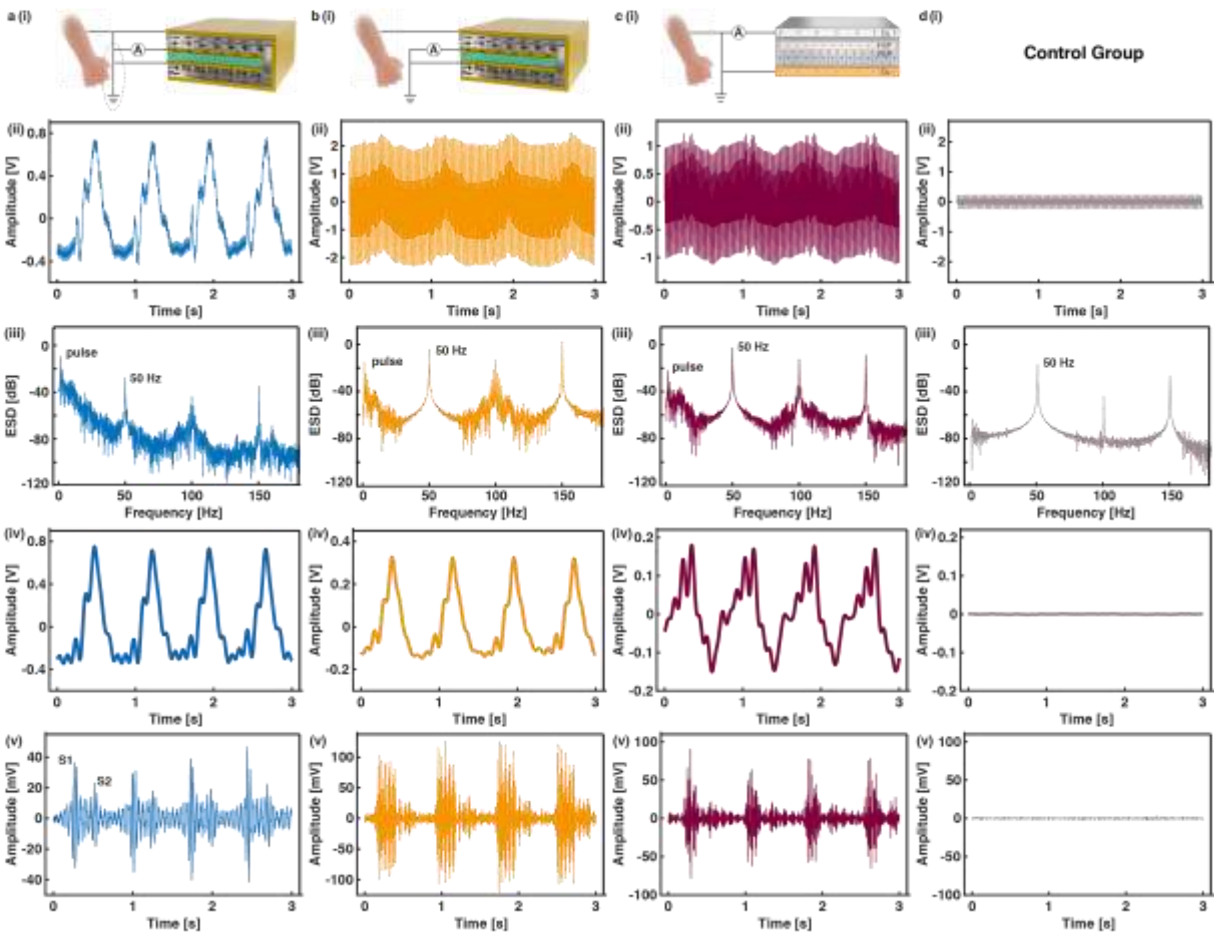

Figure S20. Improvement in SNR using the shielding layer and electrical ground. A comparative experiment involving 4 sensors has been conducted: (a, i) using the external shielding electrode and the electrical ground, (b, i) using the external shielding electrode and without the electrical ground, (c, i) using a device without the external shielding electrode, and (d, i) a basic circuit with background noises without electrodes (control group). Physiological signals at the heart apex are measured simultaneously. Comparing the (ii) original signals, and (iii) pulse signals (0-10 Hz), (iv) heart sound signals (20-200 Hz) separated by corresponding filters, it can be found that shielding and grounding significantly improve the SNR of physiological signals (especially the weak physiological signals, such as heart sounds).

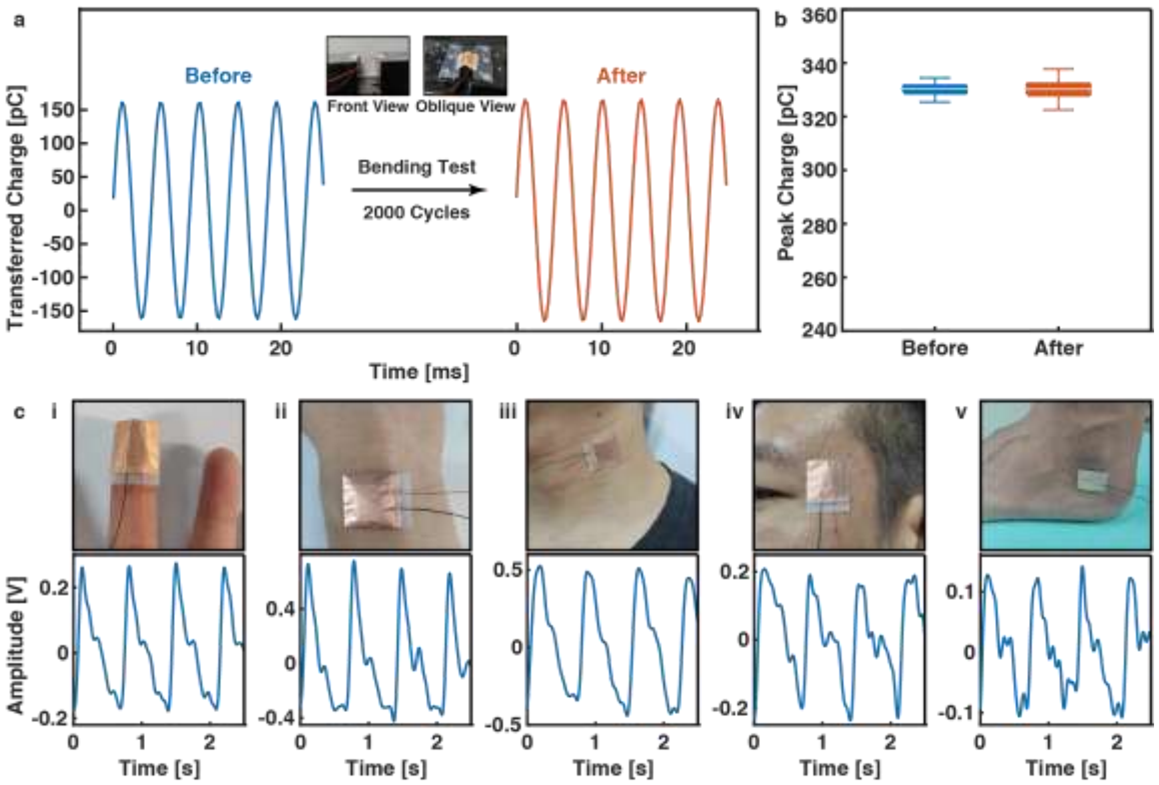

d

	Shielding Layer (Cu)	FEP Electret	PI Spacer Layer	Internal Electrodes (Cu)
Thickness (μm)	20	50	20	20
Young's Modulus (GPa)	120	0.23	3.1	120

Figure S21. Flexibility presentation and mechanical parameters of the piezoelectret sensor. (a) Bending stability. The sensor is taped on the 3-DOF displacement stage and experiences 2000 'stretching-bending-stretching' cycles. There is no significant difference between the output of before and after the bending test, verifying the good bending stability. Inset: the sensor experiences severe bending and the cross-section becomes semi-circular, indicating the good flexibility of the sensor. (b) Statistical results of the peak charges before and after the bending test under the same applied pressure (220 Hz, 1 kPa). The output charges of more than 10^5 pressure cycles are summarized as the two box-whisker plots. The five horizontal lines from top to bottom of each box are the maximum value, the 25th percentile, the median, the 75th percentile, and the minimum value. (c) The piezoelectret sensor adhered to skin surfaces with different curvatures, and the corresponding pulse waveforms, indicating the good flexibility. (d) Mechanical parameters for each layer of the piezoelectret sensor. The Young's modulus of the FEP electret with crisscross cavities is measured by a thermal analysis system (DMA1, Mettler-Toledo, LLC.). The Young's modulus of the copper tape and PI spacer layer is obtained from the datasheet provided by the seller.

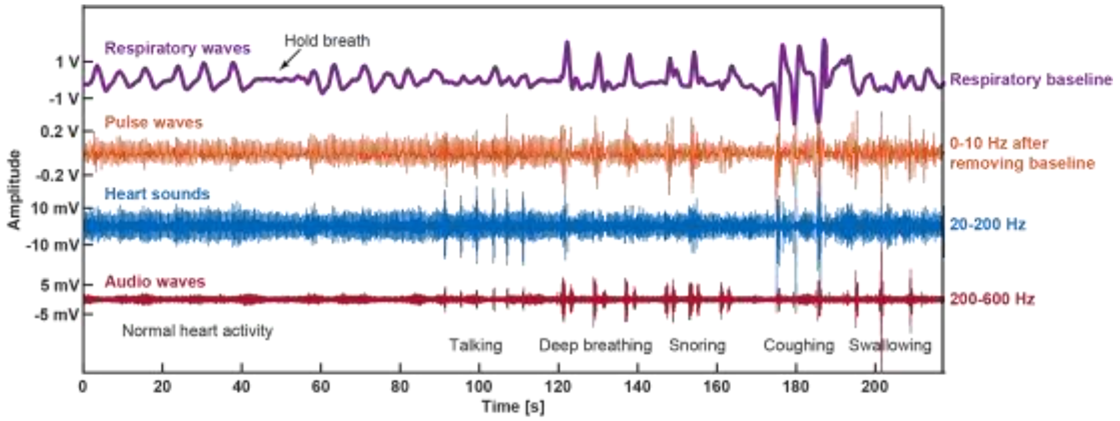

Figure S22. Continuous results of multiple physiological activities processed by filters of different frequency ranges. The 'Audio waves' in Figure 1c is actually segmental spliced after processing the original signal by different filters to highlight corresponding physiological activities. Here, the continuous filtered results with specific amplitudes are presented.

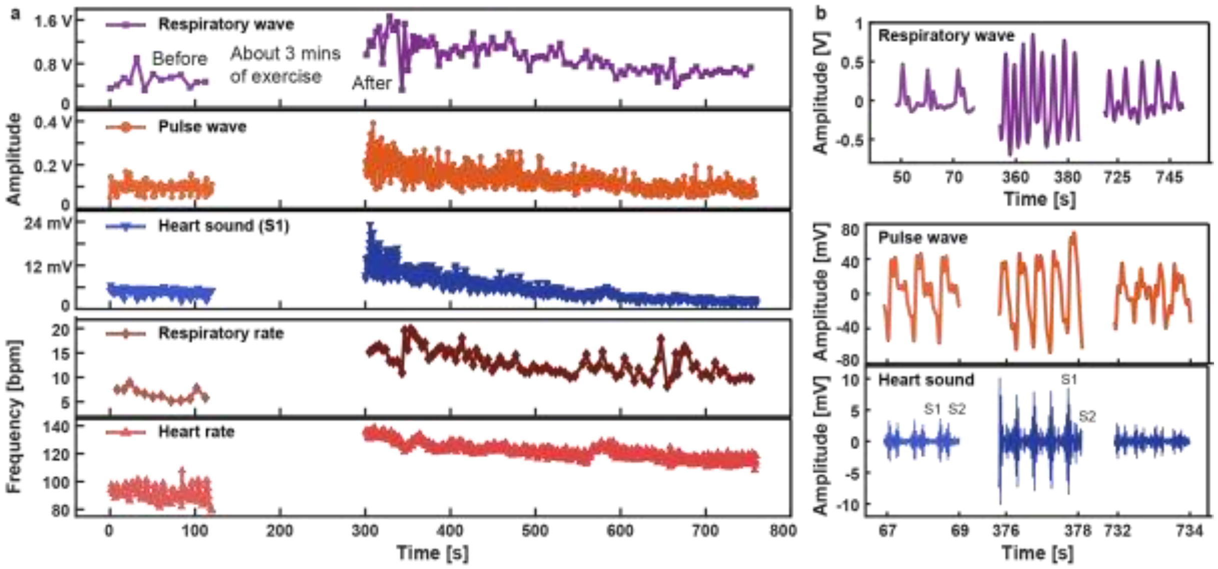

Figure S23. Effects of the exercise on respiration and cardiac activity. (a) Breathing and heart beating patterns before and after the exercise in amplitude (top) and frequency (bottom) plots. (b) The respiratory wave, pulse wave, and heart sound at specific moments (before exercise, after exercise and after rest).

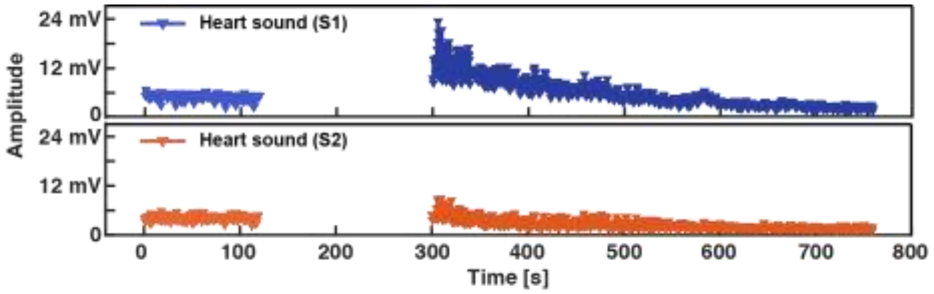

Figure S24. Comparison of the amplitude fluctuations of heart sound S1 and S2 before and after the exercise.

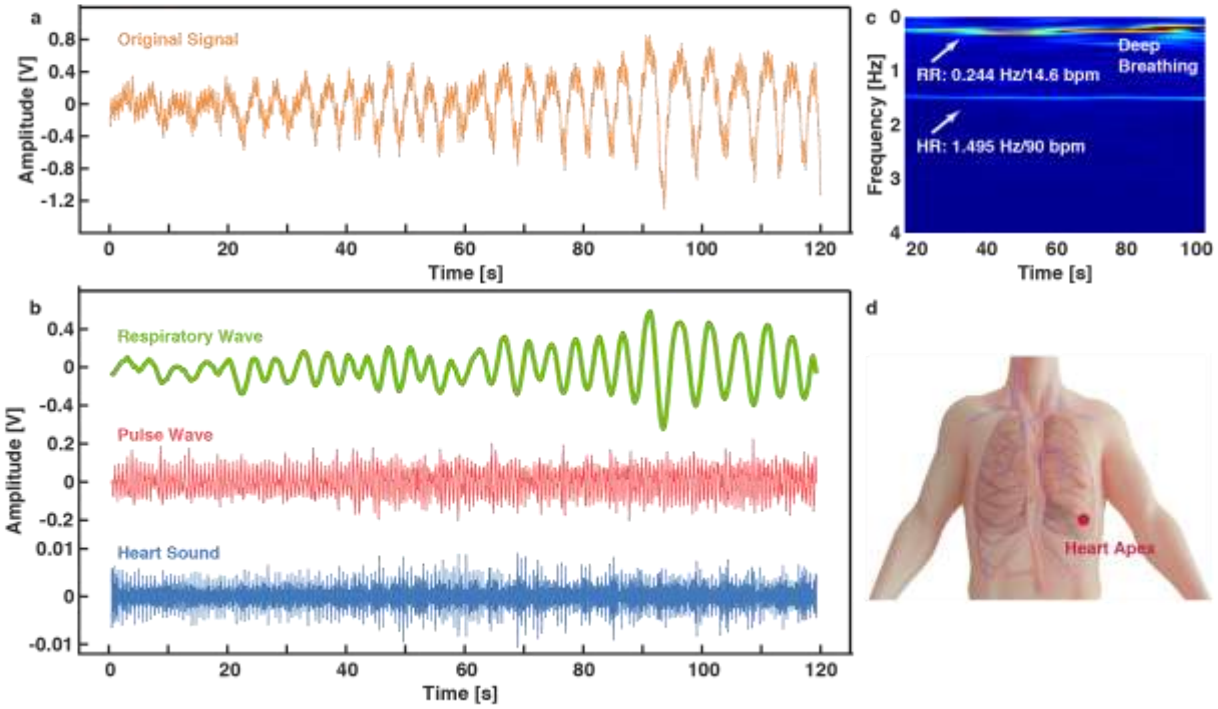

Figure S25. Time-frequency domain results under increasing respiration intensity at the heart apex. (a) Original signal and (b) filtered signals acquired from the continuous monitoring at the heart apex. (c) STFT spectrum of the original signal at the frequency range of 0-4 Hz, showing the fluctuations of heart beating and breathing. (d) Schematic diagram describing the measurement location.

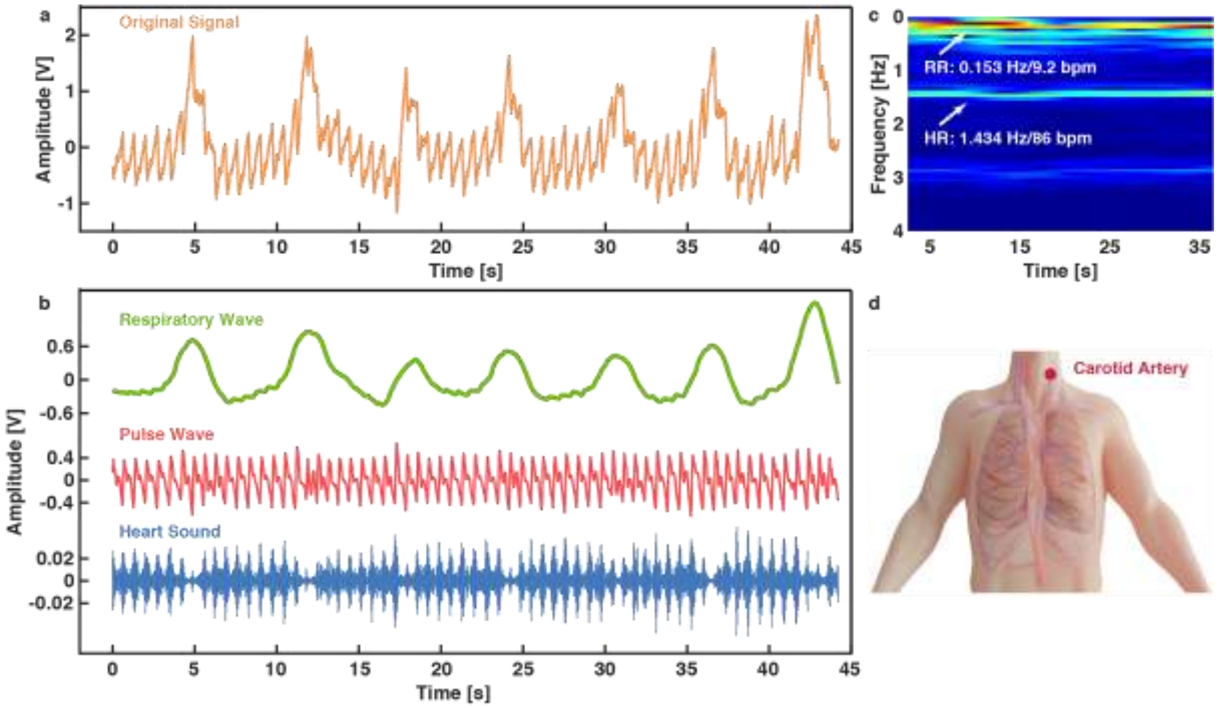

Figure S26. Time-frequency domain results at the neck. (a) Original signal and (b) filtered signals obtained at the neck. (c) STFT spectrum of the original signal at the frequency range of 0-4 Hz. (d) Schematic diagram describing the measurement location.

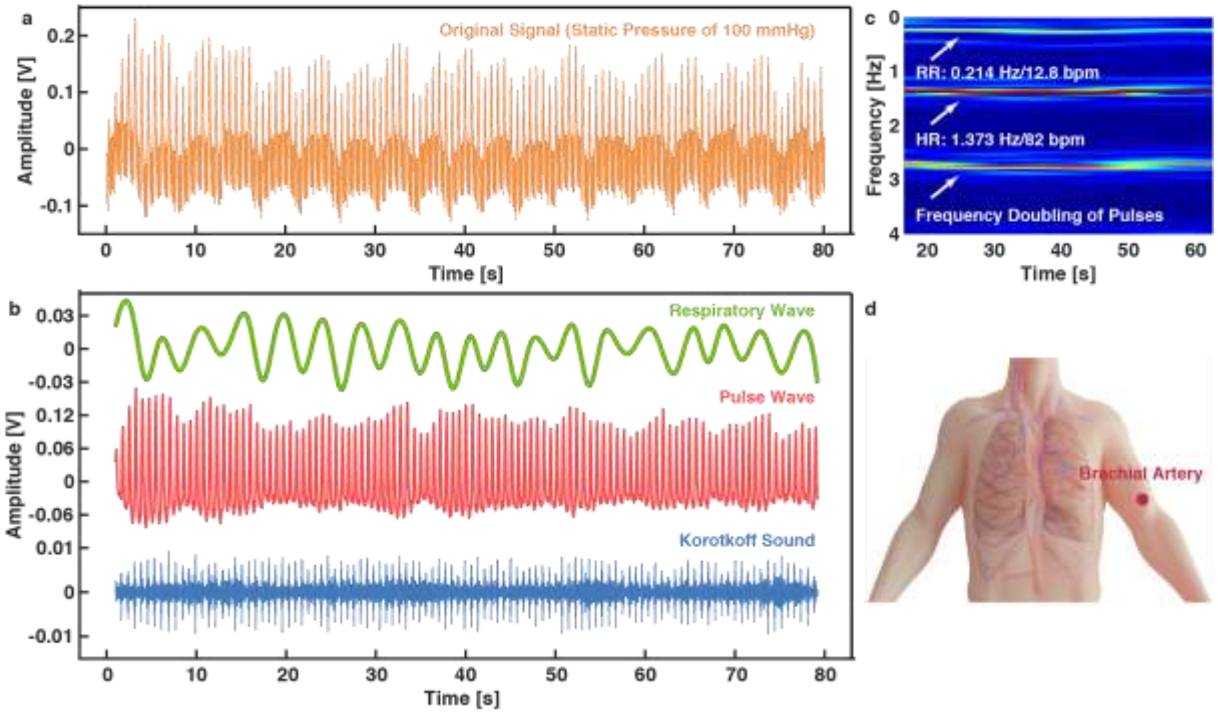

Figure S27. Time-frequency domain results at the brachial artery. (a) Original signal and (b) filtered signals obtained at the brachial artery. The sounds resulting from the heart beating at the brachial artery are also called "Korotkoff sounds". (c) STFT spectrum of the original

signal at the frequency range of 0-4 Hz. (d) Schematic diagram describing the measurement location.

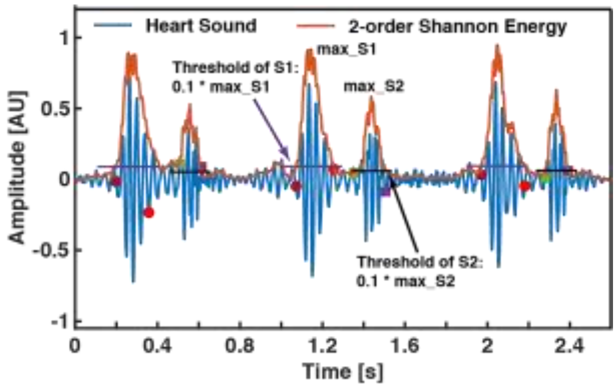

Figure S28. S1/S2 component extraction based on heart sounds segmentation. The second-order Shannon energy envelope of the heart sounds waveform is firstly calculated, and the periodic local maximas of the envelope are considered as the S1 peaks (max_S1). Then, the intersections between the threshold (0.1* max_S1) and the Shannon envelope are sought within 0.15 s before and after the S1 peaks, as the start and end points of S1 during this cardiac cycle. If there is no intersection found within 0.15 s, the point at 0.1 s before or after the S1 peak will be indicated as the start or end point of S1.

Heart sounds are divided into several cardiac cycle segments based on the S1 peaks, and the local maxima point is found for each segment as the S2 peak (max_S2). Subsequently, the process similar to S1 is conducted. That is, the intersections of the threshold (0.1* max_S2) and the Shannon envelope are found within 0.1 s before and after the S2 peak, as the start and end points of S2. If there is no intersection found within 0.1 s, the point at 0.05 s before or after the S2 peak will be forced as the start or end point of S2.

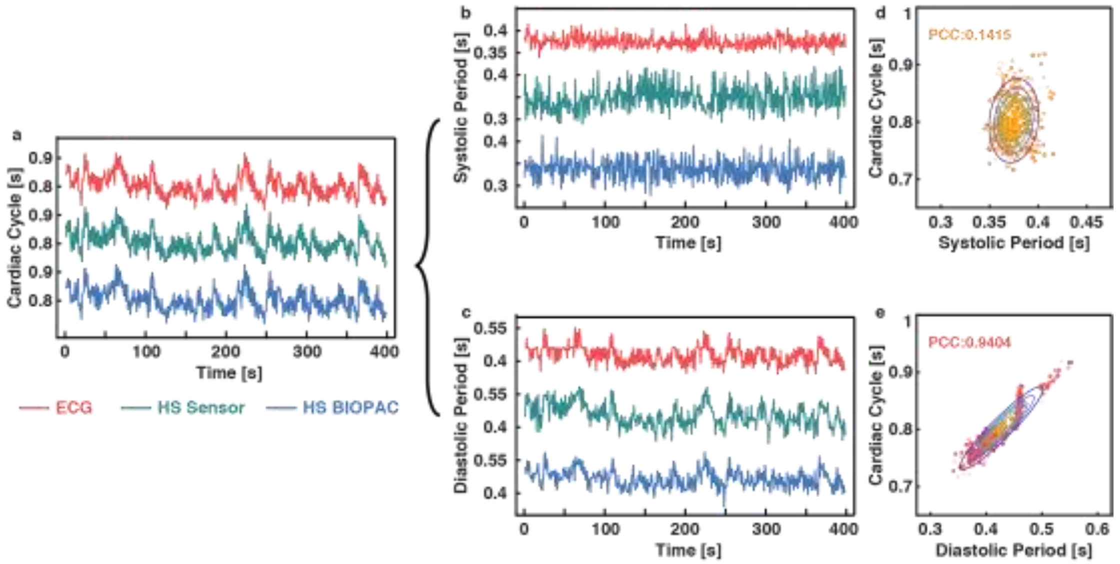

Figure S29. Further analysis of the cardiac cycle. Length of the (a) cardiac cycle, (b) systolic period and (c) diastolic period acquired from the ECG reference, piezoelectret sensor (HS Sensor) and medical physiological recorder (HS BIOPAC). Correlation between the cardiac cycle and (d) systolic period and (e) diastolic period. The length of the cardiac cycle is mainly affected by the diastolic period when the volunteer kept the resting state. PCC: Pearson correlation coefficient.

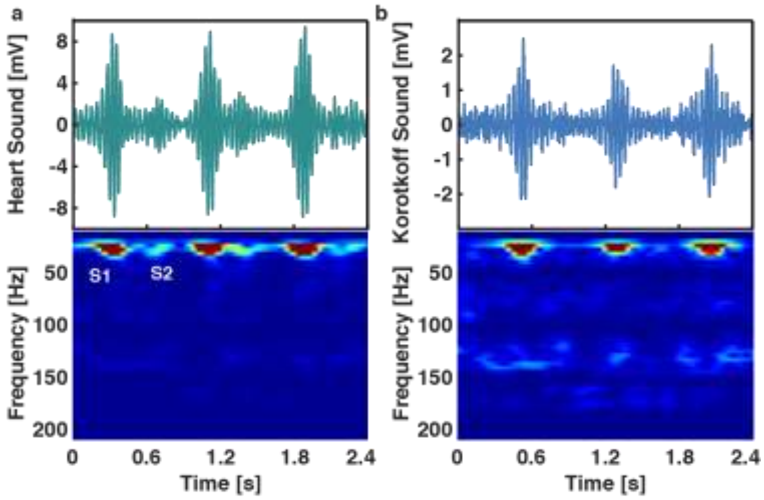

Figure S30. Simultaneously recorded (a) heart sounds and (b) Korotkoff sounds, and the corresponding STFT spectrums. For each cardiac cycle, the heart sounds contain two components, S1, S2, while the Korotkoff sounds appear to have only one identifiable component.

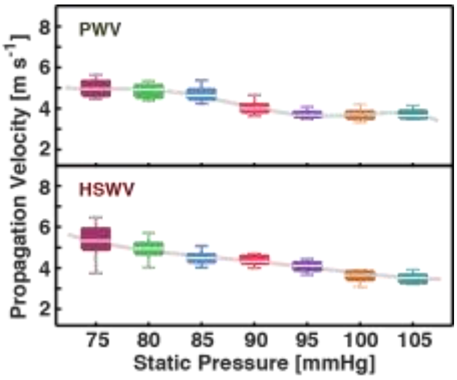

Figure S31. Fluctuation trends of PWV and HSWV under different static pressures.

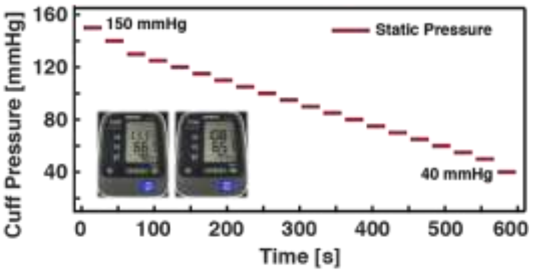

Figure S32. Applied static pressure of 150-40 mmHg by a cuff attached to a mercury manometer. Inset shows the BP reference results from Omron. BP measurement is performed twice using the Omron monitor and the mean is taken as the BP reference result.

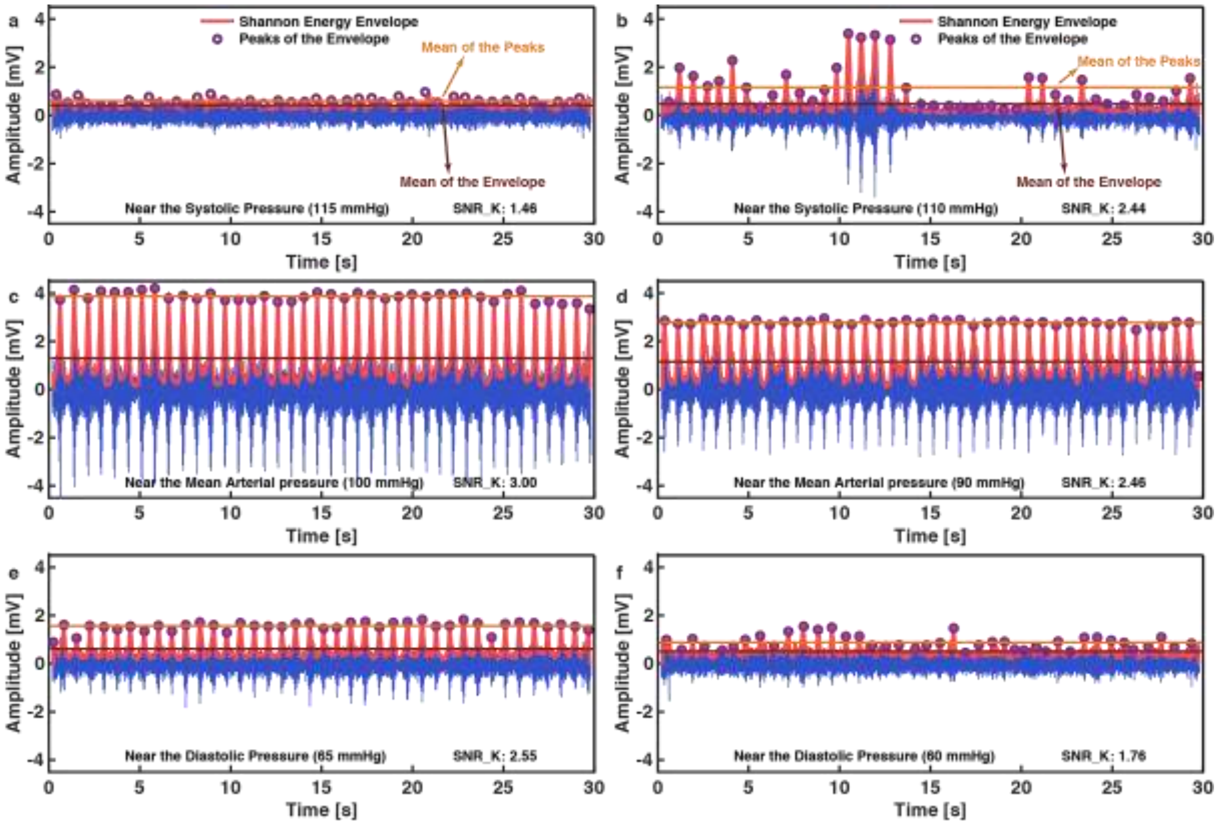

Figure S33. Calculation of the parameter SNR_K at specific pressures. (a, b) Near the systolic pressure. (c, d) Pressure between the systolic and diastolic pressure. (e, f) Near the diastolic pressure. These data are from i~vi respectively in Figure 3c.

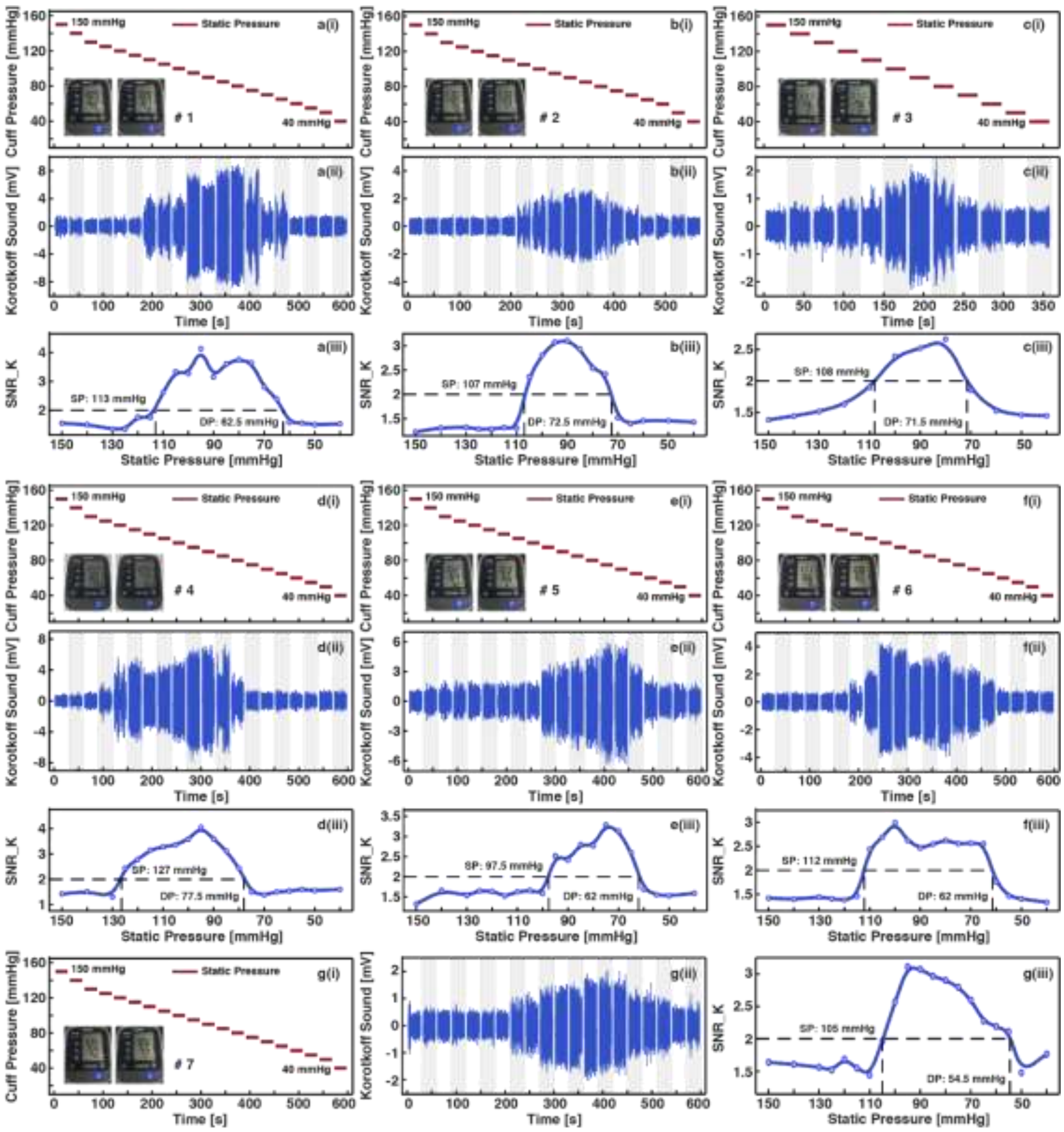

Figure S34. Detailed BP measurement results by the Korotkoff sound method for seven volunteers, with all using the same parameter threshold of SNR_K = 2. Insets show the BP results from the commercial BP monitor (Omron BP7211). BP measurement is performed using the Omron monitor before and after each Korotkoff sound method detection, and the average of the twice results is taken as the BP reference value.

Table S1. Comparison of the blood pressure results measured by the commercial blood pressure monitor (Omron BP7211) and the Korotkoff sound method (radial artery). [a)]

SBP/DBP [mmHg]	# 8	# 9
Omron [b)]	119.5/82.5	112/68
Korotkoff Sound	115.5/94.5	104/70.5
Differences	-4/12	-8/2.5

[a)] The detailed results of the two methods are shown in Figure 35 (Supporting Information);
[b)] BP measurement is performed twice for each volunteer using the Omron monitor and the mean is taken as the BP reference result.

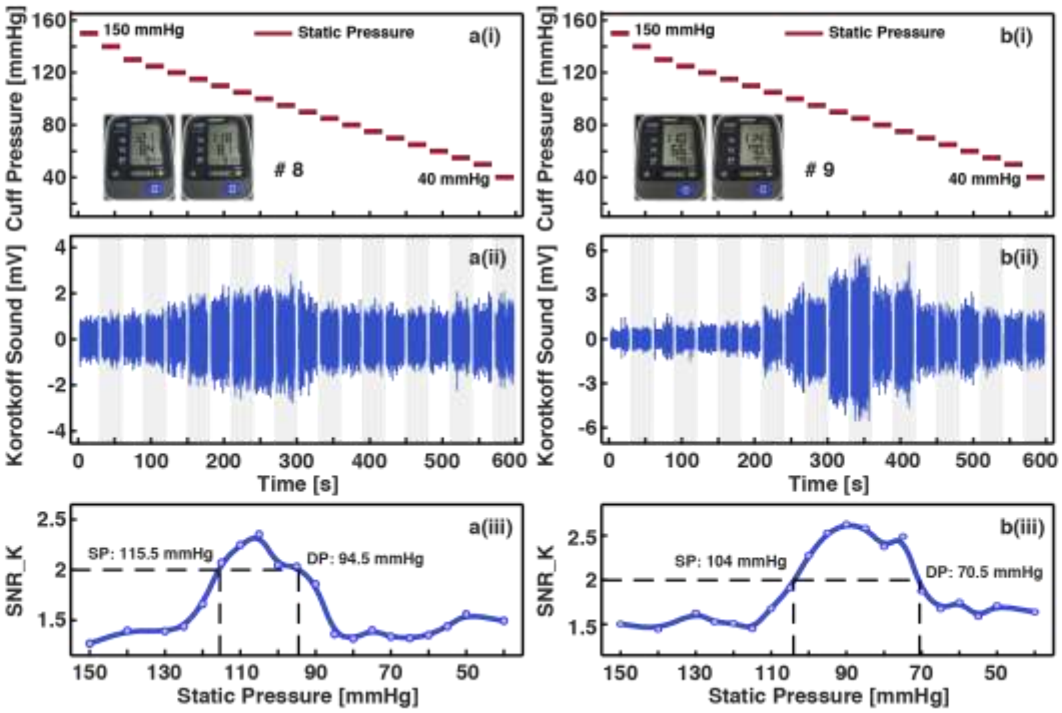

Figure S35. BP measurement results by the Korotkoff sounds at the radial artery. Insets exhibit the BP reference results from the Omron monitor.

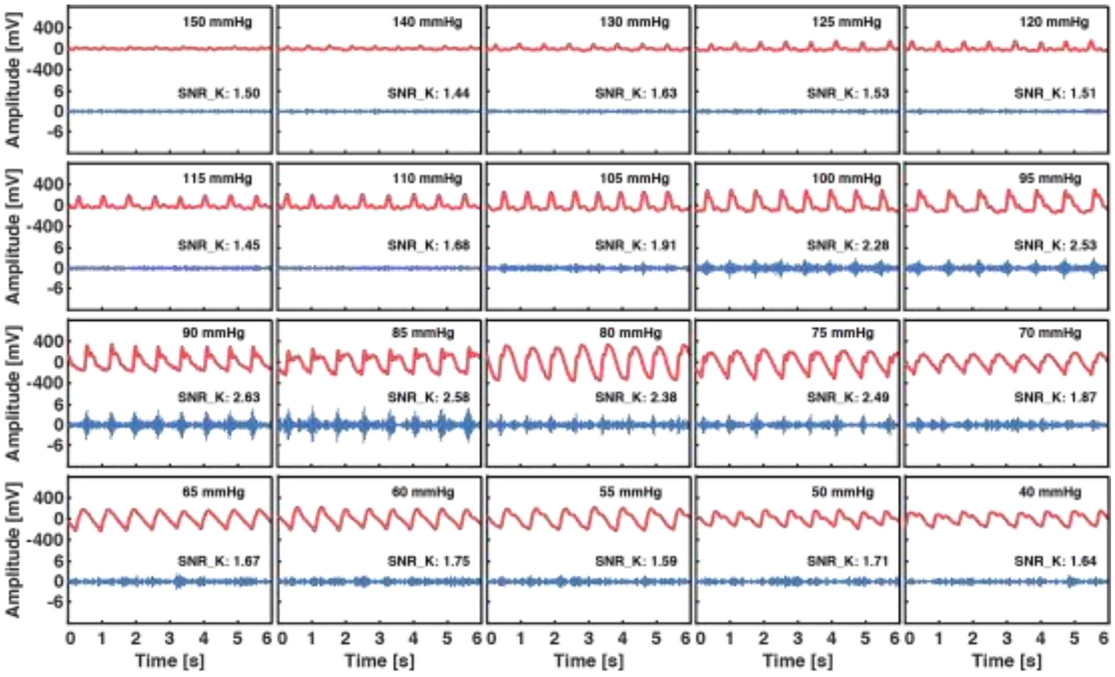

Figure S36. Detailed presentation of the radial pulses and Korotkoff sounds from the ninth volunteer (# 9).

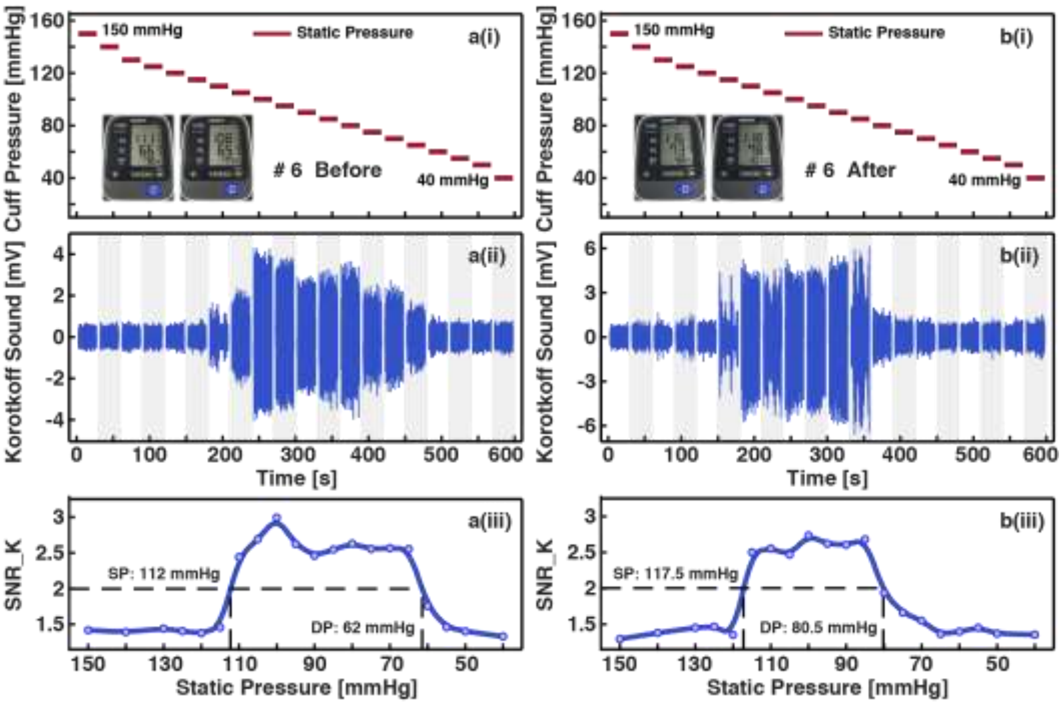

Figure S37. Comparison of the BP results (a) before and (b) after caffeine consumption for the sixth volunteer (# 6). Insets exhibit the BP reference results from the Omron monitor.

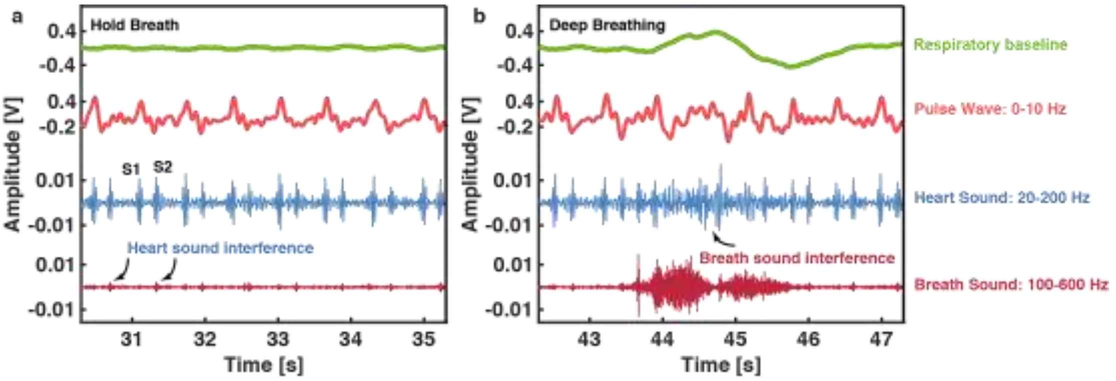

Figure S38. Enlarged view of the (a) breath holding and (b) deep breathing fragments in Figure 4a. The frequency bands of heart sounds (20-200 Hz) and breath sounds (100-600 Hz) partially overlap, and their waveforms tend to interfere with each other.

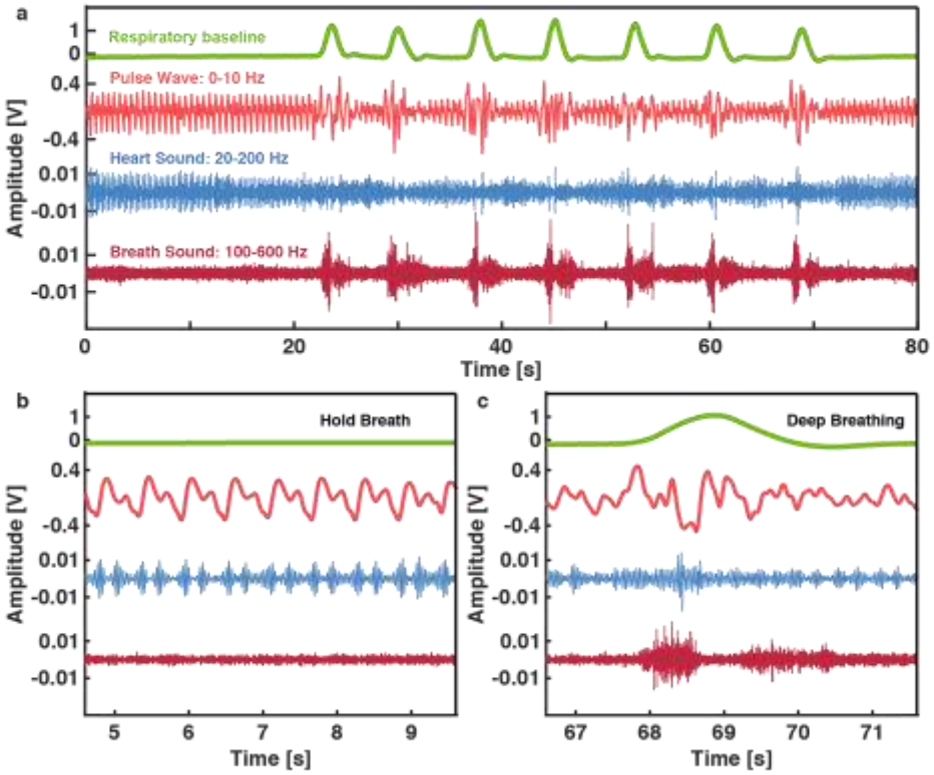

Figure S39. Breath sounds monitoring at the neck. (a) Separated physiological signals of different frequency bands from the neck during deep breathing. Enlarged view of the (b) breath holding and (c) deep breathing fragments in a.

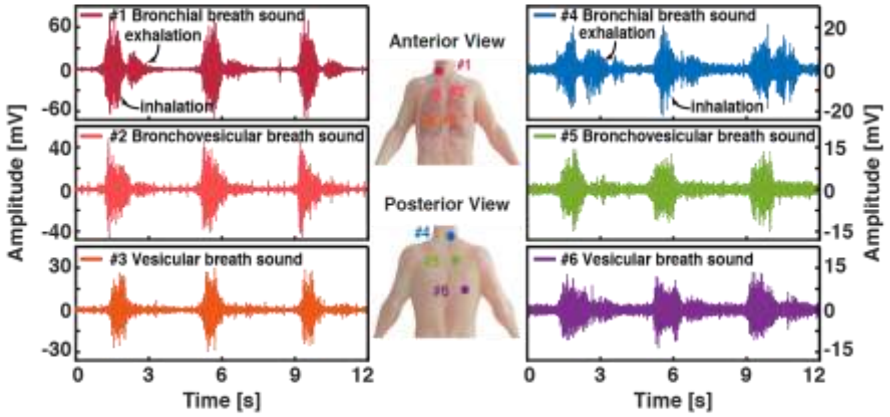

Figure S40. Summarization of time-series morphologies of the bronchial sound, bronchovesicular sound, and vesicular sound at the anterior and posterior positions for the second volunteer.

Supplementary Note 2. Classification of breath sounds

The algorithm procedure of breath sound classification is explained in this Supplementary Note. In brief, the Mel-frequency cepstral coefficients (MFCCs) of the collected breath sound are calculated and compared with those of the breath sound templates. Finally, the collected

breath sound will be classified into the category with the highest MFCCs similarity. The detailed algorithm procedure is described as follows:

1. Prepare templates for each breath sound category. In the prototype demonstration, three kinds of breath sounds are used in the classification process: normal breathing, panting after strenuous exercise, and snoring. The volunteer is asked to breathe in these three patterns to obtain the standard breath sound templates (Figure S41, Supporting Information).

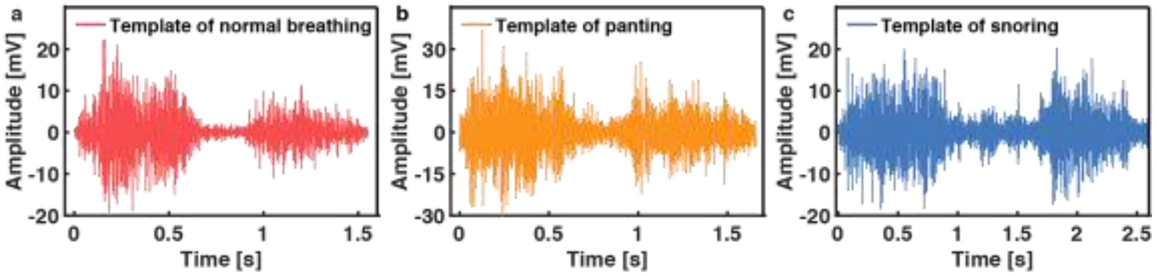

Figure S41. Prepared templates for three breathing categories of (a) normal breathing, (b) panting after strenuous exercise, and (c) snoring.

2. Calculate the MFCCs for three templates. The calculation process of MFCCs for different breath sounds is similar, and the template for normal breathing is taken as an example here to present this calculation process.

a. First, the preprocessing of the breath sound signal x(n) is conducted, including pre-emphasis and framing. Pre-emphasis is a high-pass filtering for the breath sound signal to compensate for the high-frequency components of the breath sound suppressed by fat and skin tissues. The high-pass filter used in this work is:

$$F(z) = 1 - 0.9375z^{-1} \qquad \text{(S1)}$$

Framing is the division of the breath sound signal x(n) into L short segments, $\{x_1(n), x_2(n), \ldots x_L(n)\}$, each of which can be approximately considered as the stationary signal (Figure S42a, Supporting Information). The frame length is generally 20~30 ms in speech recognition while 60 ms in this work since the variations of breath sounds are far lower than those of speech. The overlap length between adjacent frames is 20 ms.

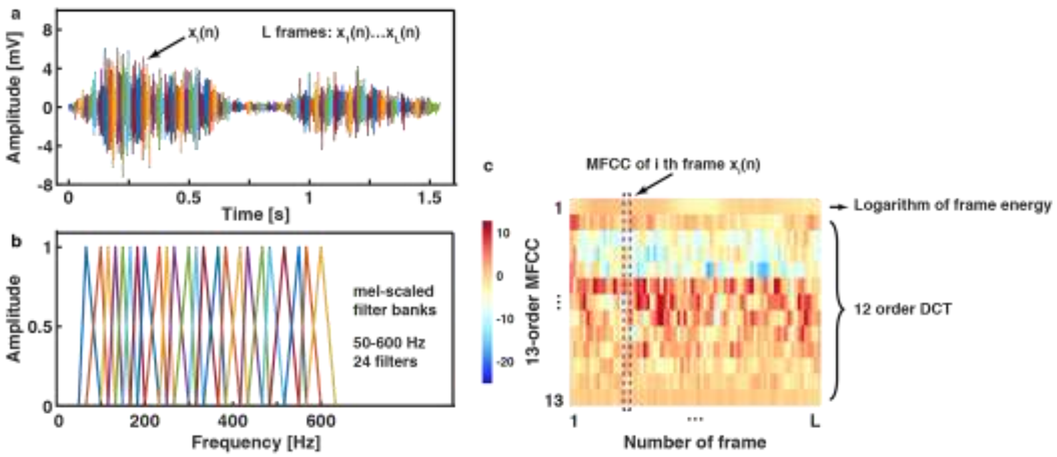

Figure S42. Calculation process of MFCCs. (a) Framing of the pre-emphasis breath sound signal. Different colors represent different frames. The frame length is 60 ms and the overlap between adjacent frames is 20 ms. (b) Designed mel-scale filter banks. 24 mel-scale filters are designed within 50-600 Hz. (c) Calculated MFCCs results. The MFCCs of each breath sound frame are composed of the logarithmic energy and 12-order DCT.

b. Mel-scale filter banks is designed. Mel-scale filter banks are composed of a series of nonlinear triangular band-pass filters, which are constructed so that the lower limit of one filter is located at the center frequency of the previous filter, while the upper limit of the same filter is located at the center frequency of the next filter. The relationship between the mel-scale frequency and the real linear-scale frequency can be written as:

$$f_{mel} = 1125 \ln(1 + \frac{f_{lin}}{700}) \quad \text{(S2)}$$

where f_{mel} and f_{lin} are the mel-scale and the linear-scale frequency, respectively. Finally, 24 mel-scale filters are designed within 50-600 Hz given the frequency bandof breath sounds (Figure S42b, Supporting Information), which are expressed as $\{H_1(k), H_2(k) \ldots H_{24}(k)\}$ in the frequency domain.

c. For each breath sound frame $x_i(n)$, the logarithmic energy corresponding to each mel-scale filter after windowing is calculated. The window function used is a hamming window (w(n)) of equal length to the breath sound frame. If $|X_i(k)|$ is the FFT result of the windowed breath sound frame $x_i(n)w(\text{n})$, $|X_i(k)|^2$ is the energy spectrum. Then, the logarithmic energy corresponding to each mel-scale filter can be expressed as:

$$E(m) = \ln(\sum_{k=0}^{N-1} |X_i(k)|^2 H_m(k)), 1 \le m \le 24 \quad \text{(S3)}$$

where N is the length of the FFT spectrum $|X_i(k)|$ (or the filter $H_m(k)$).

d. The discrete cosine transform (DCT) of the logarithmic energy is calculated as:

$$C(a) = \sum_{m=1}^{24} E(m) \cos(\frac{a(m-0.5)\pi}{24}), 1 \le a \le T \quad \text{(S4)}$$

where a is the order of the calculated DCT , and its value is between 1 and T. T is chosen as 12 since the DCT result is symmetric about the normalized frequency π in 0~2π. Finally, 12-order DCT results are obtained: $\{C(1);\ C(2); \dots\ C(12)\}$.

e. The total logarithmic energy of each breath sound frame can be written as:

$$C(0) = \ln(\sum_{k=0}^{N-1} |X_i(k)|^2) \qquad \text{(S5)}$$

The 13-order MFCCs can be expressed as:

$$MFCC(i) = \{C(0);\ C(1);\ C(2); \dots\ C(12)\} \qquad \text{(S6)}$$

The calculation process in 2a~2e is repeated for each breath sound frame to obtain the corresponding MFCCs results, and the MFCCs of the entire breath sound can be expressed as $\{MFCC(1);\ MFCC(2); \dots\ MFCC(L)\}$ (Figure S42c, Supporting Information).

f. In some applications, the first-order and second-order difference of the 13-order MFCCs are further calculated to obtain 39-order MFCCs. However, the 13-order MFCCs are sufficient for the breath sound classification in this work, so the 39-order MFCCs is not calculated to avoid data redundancy. Finally, the MFCCs of the three breath sound templates (‘template_n’ for the normal breathing, ‘template_p’ for the panting, and ‘template_s’ for the snoring) are presented in Figure S43, Supporting Information.

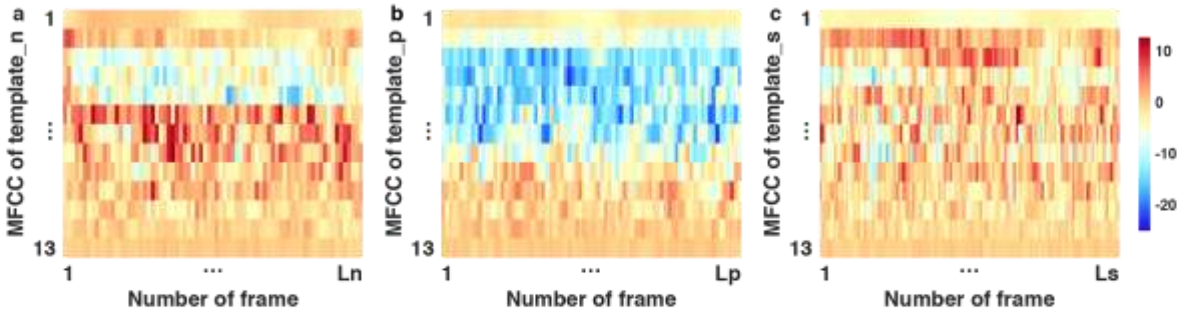

Figure S43. MFCCs of three breath sound templates of (a) normal breathing, (b) panting, and (c) snoring.

3. For the collected unknown breath sound, the process in 2a~2e is repeated to calculate the corresponding MFCCs (Figure S44a, Supporting Information).

4. The similarity between the MFCCs of unknown breath sound and those of three templates is compared, and the unknown breath sound is classified into the category with the highest similarity (Figure S44b, Supporting Information). For quantitative analyses of the similarity, a parameter, σ_h^2/σ_v^2, is introduced after mapping the MFCCs results of two breath sounds into a scatter plot, where σ_h^2 and σ_v^2 are the variances of results along the parallel and perpendicular direction to the diagonal axis ($y = x$), respectively. A two-dimensional Gaussian distribution

is used to fit these mapped points, and σ_h^2 and σ_v^2 are obtained (Figure S44c, Supporting Information). A large σ_h^2/σ_v^2 value indicates high similarity, while a small value means high dissimilarity since points from two sets of identical values would be mapped to the diagonal axis ($y = x$) to result in the σ_h^2/σ_v^2 value of infinite. Therefore, the maximum value in $\{\sigma_{h\_n}^2/\sigma_{v\_n}^2, \sigma_{h\_p}^2/\sigma_{v\_p}^2, \sigma_{h\_s}^2/\sigma_{v\_s}^2\}$ is found and the unknown breath sound is classified into the corresponding category (Figure S44d, Supporting Information).

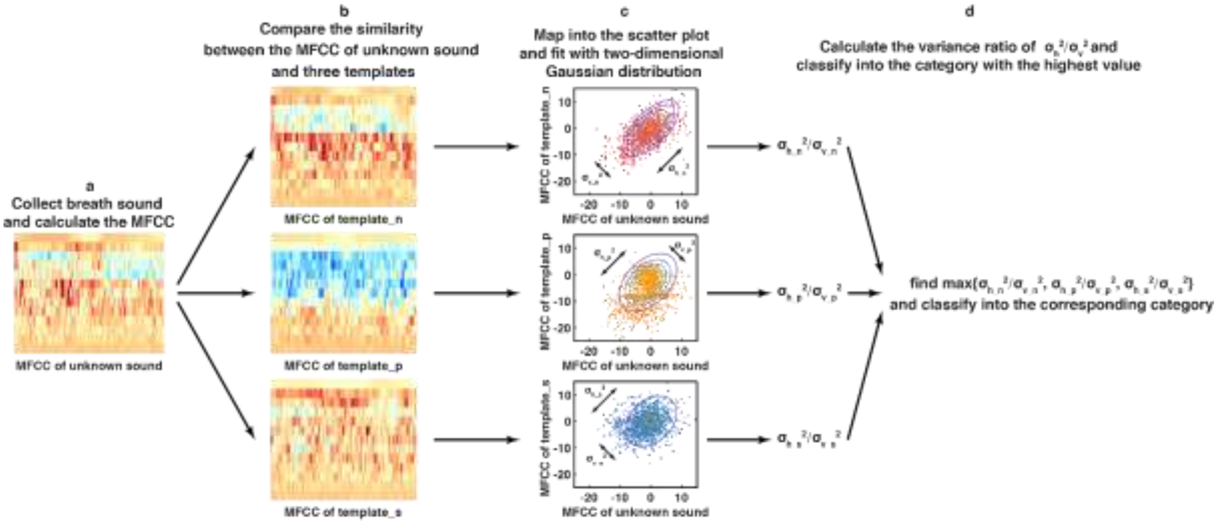

Figure S44. Algorithm procedure of the breath sound classification. (a) Unknown breath sound is collected and the MFCCs are calculated. (b) The similarity between the MFCCs of unknown breath sound and those of three templates is compared. (c) The parameter σ_h^2/σ_v^2 is introduced to quantify the similarity. After the MFCCs results of two breath sounds are mapped into the scatter plot, the two-dimensional Gaussian distribution is used to fit these mapped points, and the variances along the directions parallel to and perpendicular to the diagonal axis are obtained. (d) The variance ratio between the unknown breath sound and the three templates is calculated and the maximum value is found. The unknown breath sound will be classified into the corresponding category with the highest σ_h^2/σ_v^2 value.

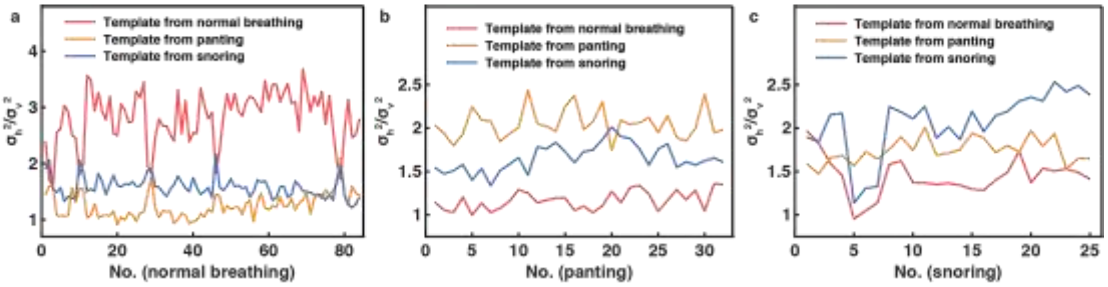

Figure S45. Original results of the parameter σ_h^2/σ_v^2 between the collected breath sounds and three templates. (a) 84 cases of normal breathing. (b) 32 cases of panting. (c) 25 cases of

snoring. Before the formal classification process, a template was prepared for each kind of breath sounds. The σ_h^2/σ_v^2 results between each collected breath sound and the three templates are calculated separately, and the collected breath sound will be classified into the category with the highest parameter value.

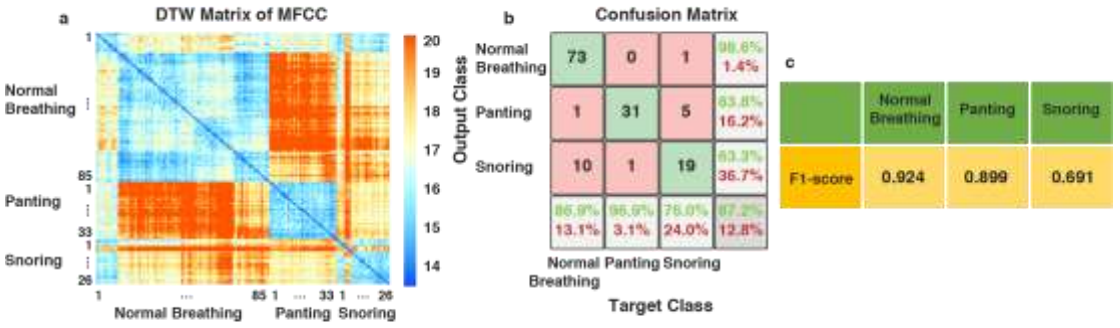

Figure S46. Breath sounds classification by DTW algorithm. (a) DTW matrix of MFCC from different breath sounds (containing the three templates corresponding to each kind of breath sounds). A small DTW distance (blue color) means a high similarity, while a large DTW distance (orange color) indicates a high dissimilarity. Three blue squares along the diagonal line illustrate high similarity among the breath sounds from the same pattern. (b) Confusion matrix of the classification results using the DTW distance. (c) F1-scores for the three breathing patterns.

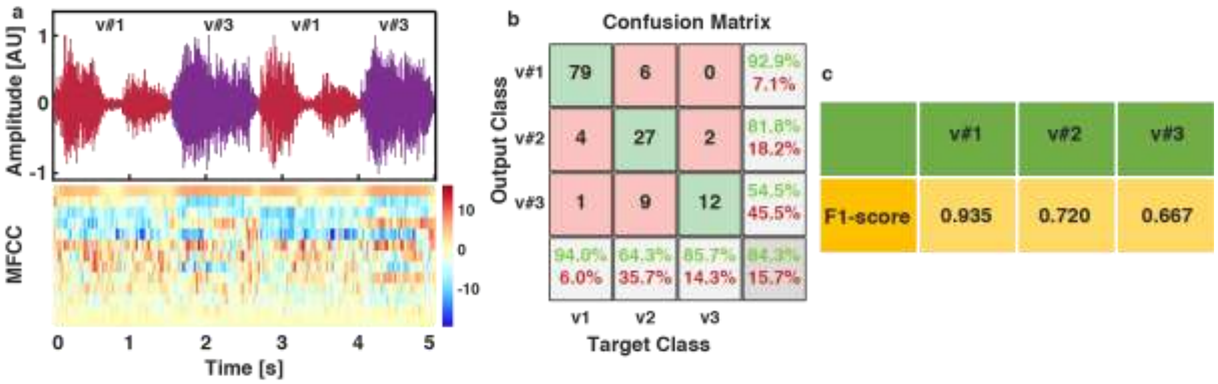

Figure S47. Breath sounds classification for human identity recognition. Breath sounds from three volunteers (84 cases for v#1, 42 cases for v#2, and 14 cases for v#3) participated in the classification process. (a) Presentation of distinguishing different volunteers through MFCC. (b) Confusion matrix of the classification results. (c) F1-scores for the three volunteers.

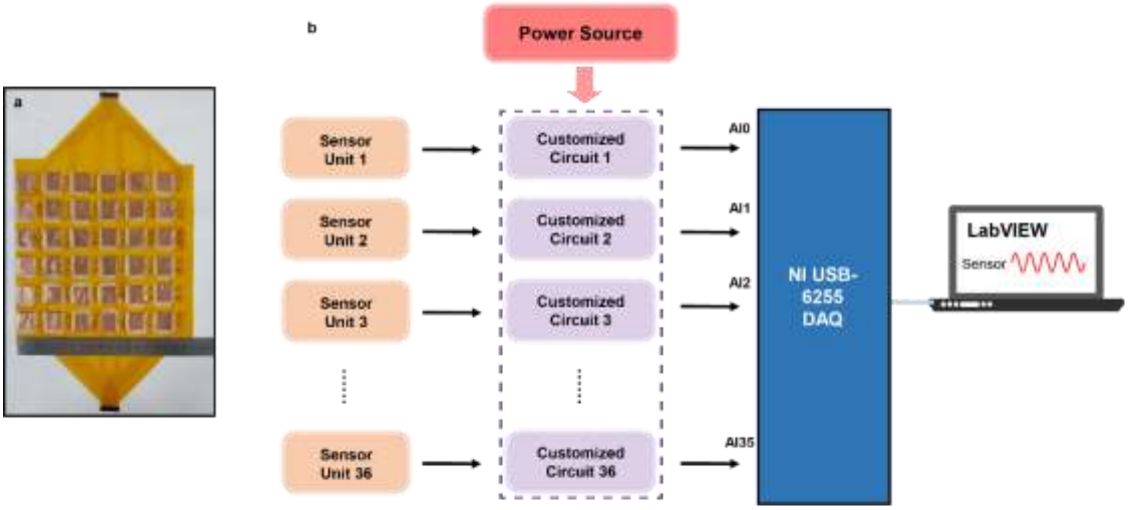

Figure S48. Heart sounds sensor array and data acquisition system. (a) Photographic image of the 6×6 sensor array adhered on a flexible printed circuit board (fPCB). (b) Data acquisition system for the sensor array. The output signal of each sensor unit is processed by the corresponding customized circuit (Figure S8) and collected by NI USB-6255 in the differential mode. Independent structure design and circuit composition ensure the low crosstalk among the 36 sensor units.

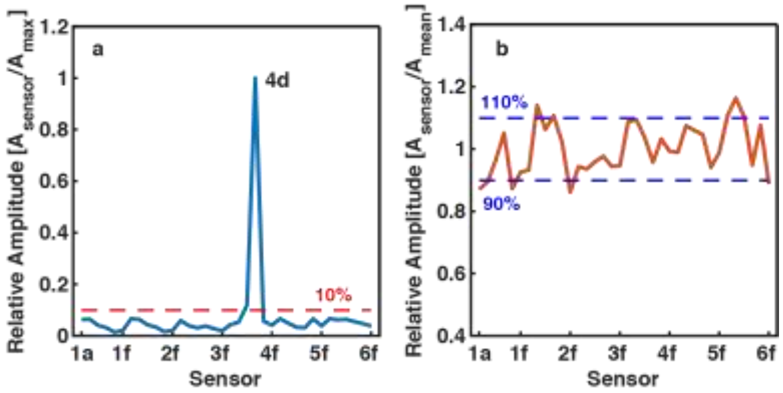

Figure S49. (a) Crosstalk and (b) consistency characterization of the 6×6 sensor array.

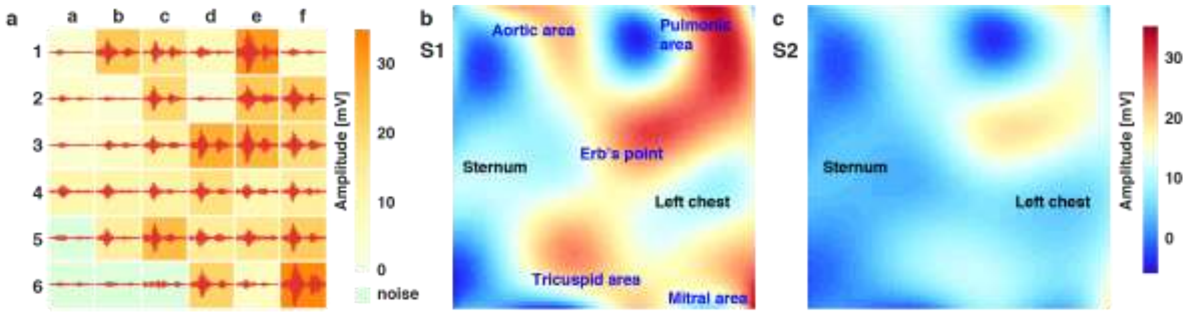

Figure S50. Measurement of the heart sounds distribution for the same volunteer using the medical physiological recorder (BIOPAC®). (a) Summarization of the heart sounds results

acquired from BIOPAC®. Mapping distributions of (b) S1 and (c) S2 intensities. The original 6×6 results were smoothed by cubic spline interpolation to highlight the position-specific amplitude differences.

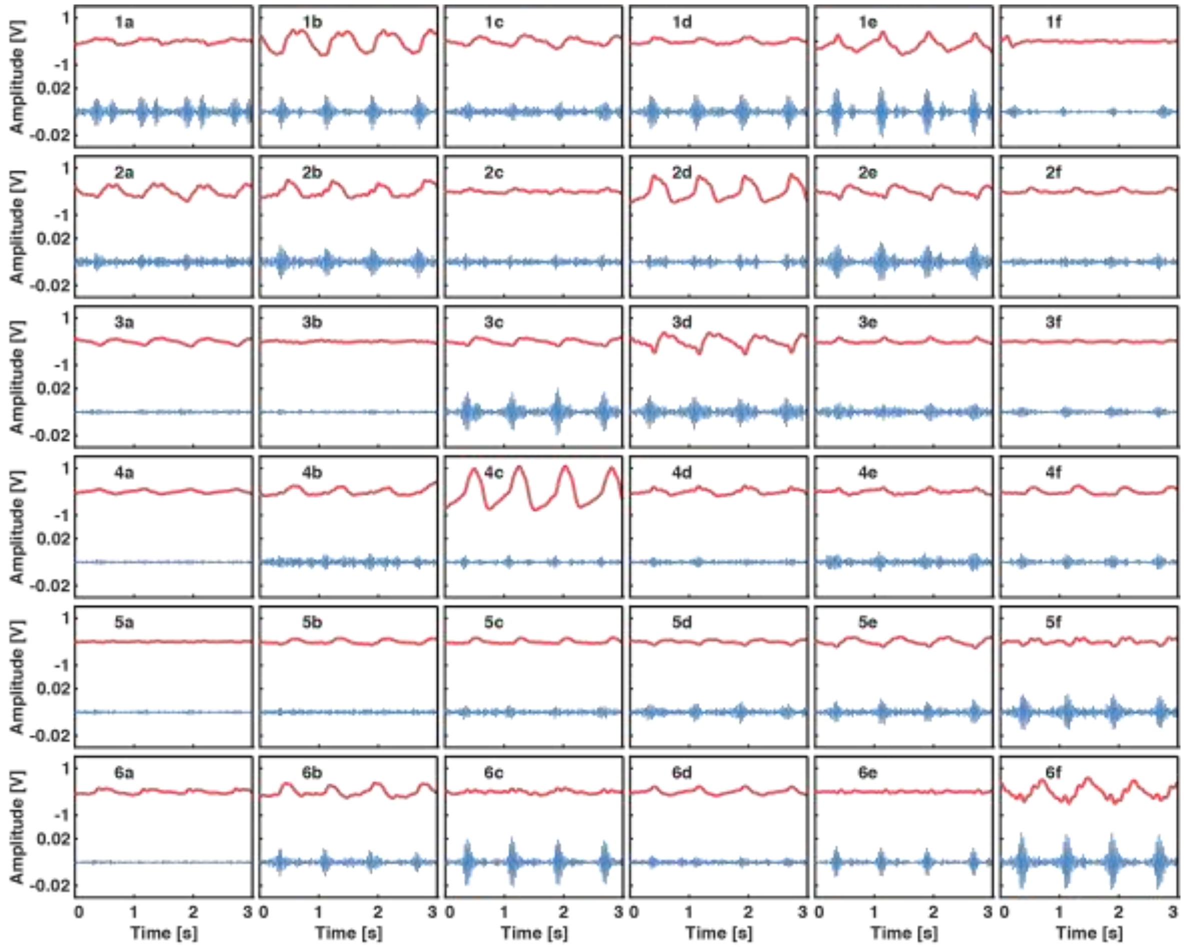

Figure S51. Mapping distribution of the heart sounds and pulses measured simultaneously from the 6×6 sensor array.

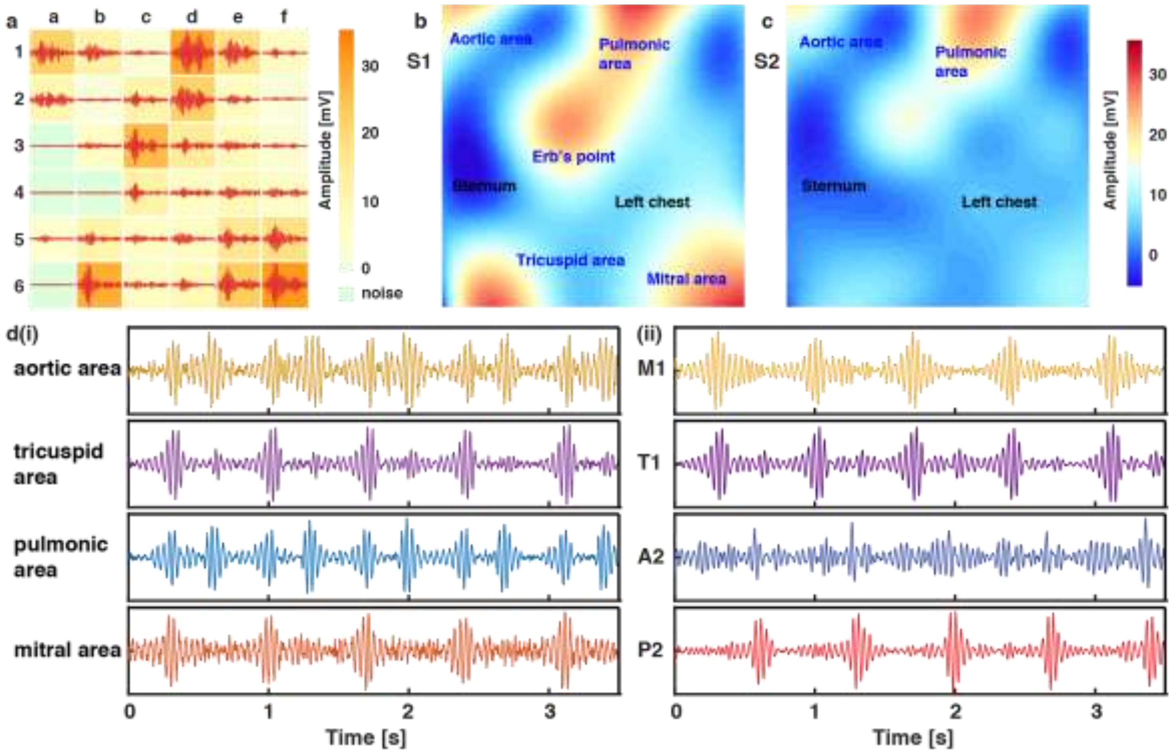

Figure S52. Heart sounds mapping and unmixing from the second volunteer. (a) Summarization of the heart sounds results acquired from the 36 sensor units. Mapping distributions of (b) S1 and (c) S2 intensities. The original 6×6 results were smoothed by cubic spline interpolation to highlight the position-specific amplitude differences. (d) Demonstration of heart sounds separation by ICA. (i) Original heart sounds from the four valve areas. (ii) Corresponding four valve components after the ICA unmixing.
